# A streptococcal Fic domain-containing protein disrupts blood-brain barrier integrity by activating moesin in endothelial cells

**DOI:** 10.1371/journal.ppat.1007737

**Published:** 2019-05-09

**Authors:** Zhe Ma, Jie Peng, Dandan Yu, Joseph S. Park, Huixing Lin, Bin Xu, Chengping Lu, Hongjie Fan, Matthew K. Waldor

**Affiliations:** 1 MOE Joint International Research Laboratory of Animal Health and Food Safety, College of Veterinary Medicine, Nanjing Agricultural University, Nanjing, Jiangsu, China; 2 Division of Infectious Diseases, Brigham and Women’s Hospital, Harvard Medical School, Boston, Massachusetts, United States of America; 3 Department of Microbiology, Harvard Medical School, Boston, Massachusetts, United States of America; 4 Ministry of Agriculture Key Laboratory of Animal Bacteriology, Nanjing, Jiangsu, China; 5 Jiangsu Co-innovation Center for Prevention and Control of Important Animal Infectious Diseases and Zoonoses, Yangzhou, Jiangsu, China; 6 Howard Hughes Medical Institute, Boston, Massachusetts, United States of America; INSERM, FRANCE

## Abstract

*Streptococcus equi* subsp. *zooepidemicus* (SEZ) is a zoonotic pathogen capable of causing meningitis in humans. The mechanisms that enable pathogens to traverse the blood-brain barrier (BBB) are incompletely understood. Here, we investigated the role of a newly identified Fic domain-containing protein, BifA, in SEZ virulence. BifA was required for SEZ to cross the BBB and to cause meningitis in mice. BifA also enhanced SEZ translocation across human Brain Microvascular Endothelial Cell (hBMEC) monolayers. Purified BifA or its Fic domain-containing C-terminus alone were able to enter into hBMECs, leading to disruption of monolayer barrier integrity. A SILAC-based proteomic screen revealed that BifA binds moesin. BifA’s Fic domain was required for its binding to this regulator of host cell cytoskeletal processes. BifA treatment of hBMECs led to moesin phosphorylation and downstream RhoA activation. Inhibition of moesin activation or moesin depletion in hBMEC monolayers abrogated BifA-mediated increases in barrier permeability and SEZ’s capacity to translocate across monolayers. Thus, BifA activation of moesin appears to constitute a key mechanism by which SEZ disrupts endothelial monolayer integrity to penetrate the BBB.

## Author summary

*Streptococcus equi* subsp. *zooepidemicus* (SEZ) is an important animal pathogen and can cause meningitis in humans. Little is known about how this Group C streptococcal species penetrates the blood-brain barrier (BBB). We identified *bifA*, a gene that is critical for SEZ to cause meningitis in mice and to penetrate a human brain endothelial monolayer in a tissue culture model. BifA’s Fic domain enables the protein to enter into endothelial monolayers and to bind to moesin, a cytoskeletal regulatory protein, leading to its activation. Preventing moesin activation abolished BifA-induced barrier leakiness and SEZ’s capacity to penetrate a monolayer barrier. Together, our findings suggest that SEZ meningitis depends on BifA, a Fic-domain protein that manipulates moesin-dependent signaling to modulate BBB permeability.

## Introduction

*Streptococcus equi* subsp. *zooepidemicus* (SEZ) is a Lancefield Group C opportunistic pathogen capable of infecting a broad range of animal species, including humans [1]. The most significant burden of disease caused by SEZ is in farmed animals, including horses, cows and pigs [2]. However, human SEZ infections have been reported globally and are often linked to consumption of unpasteurized milk or contact with infected animals. Meningitis is the most common clinical manifestation of human infection with SEZ and can be fatal [3, 4].

SEZ, like most streptococci, is an extracellular pathogen [2] and to cause meningitis, these organisms must penetrate the blood-brain barrier (BBB), a functional barrier established in part by the endothelial cells lining the brain microvasculature. This highly selective barrier between the brain and the circulatory system acts as an important protective mechanism, excluding blood-borne pathogens and toxins from the central nervous system [5]. While relatively high pathogen concentrations in blood are thought to be a prerequisite for organisms to traverse the BBB, different pathogens appear to rely on varied mechanisms to penetrate this barrier [5]. Diverse factors facilitating pathogen adhesion to brain capillary endothelial cells have been identified and both transcellular and paracellular routes for pathogens to cross the BBB have been reported [6, 7].

Although SEZ virulence factors that facilitate pathogen adhesion to host tissue and immune evasion have been identified [8–10], there is little knowledge of the factors and mechanisms that enable SEZ to penetrate the BBB. In previous research, we sequenced and compared the genome sequence of a virulent SEZ strain (ATCC35246, isolated from a dead pig) to those of non-virulent SEZ strains, to identify potential virulence-linked genes [11]. Several loci in the ATCC35246 isolate appeared to have been acquired through horizontal gene transfer. One such region (pathogenicity island II) contained a gene (SeseC_01334) that is predicted to encode a protein carrying an N-terminal RhuM domain and a C-terminal Fic domain. These two domains are linked to virulence in other pathogens. Fic (filamentation induced by cyclic AMP) domain-containing proteins are present in many animal and plant pathogens [12]. Often these proteins are delivered via type III or type IV secretion systems (T3SS, T4SS) directly into the cytosol of host cells, where they manipulate host signaling pathways via covalent modification of target proteins. Though Fic proteins induce varied modifications in their targets (e.g., AMPylation, UMPylation, phosphorylation and phosphocholination have been described), they all share a consensus 9 amino acid core, HxFx(D/E)(A/G)N(K/G)R, with the histidine residue exhibiting the greatest conservation [12]. Since Fic domain proteins are linked to pathogenicity, we investigated whether SeseC_01334 (here re-named BifA, for brain invasion factor) contributes to SEZ virulence.

We show that BifA is critical for SEZ to disrupt the BBB and to infect the mouse brain. Furthermore, this Fic-domain protein is required for SEZ to penetrate a tissue culture model of the BBB. BifA’s Fic domain enables the protein to enter into and to disrupt the barrier function of brain endothelial monolayers. BifA targets moesin and leads to its phosphorylation. Inhibition of moesin phosphorylation or knockdown of moesin expression prevented BifA-mediated increases in monolayer permeability and SEZ’s capacity to penetrate a monolayer barrier. Collectively, our findings reveal that SEZ meningitis depends on BifA, a Fic-domain protein that disrupts BBB function by manipulating moesin-dependent signaling.

## Results

### *BifA* augments SEZ virulence and promotes BBB penetration

**Fig 1:**
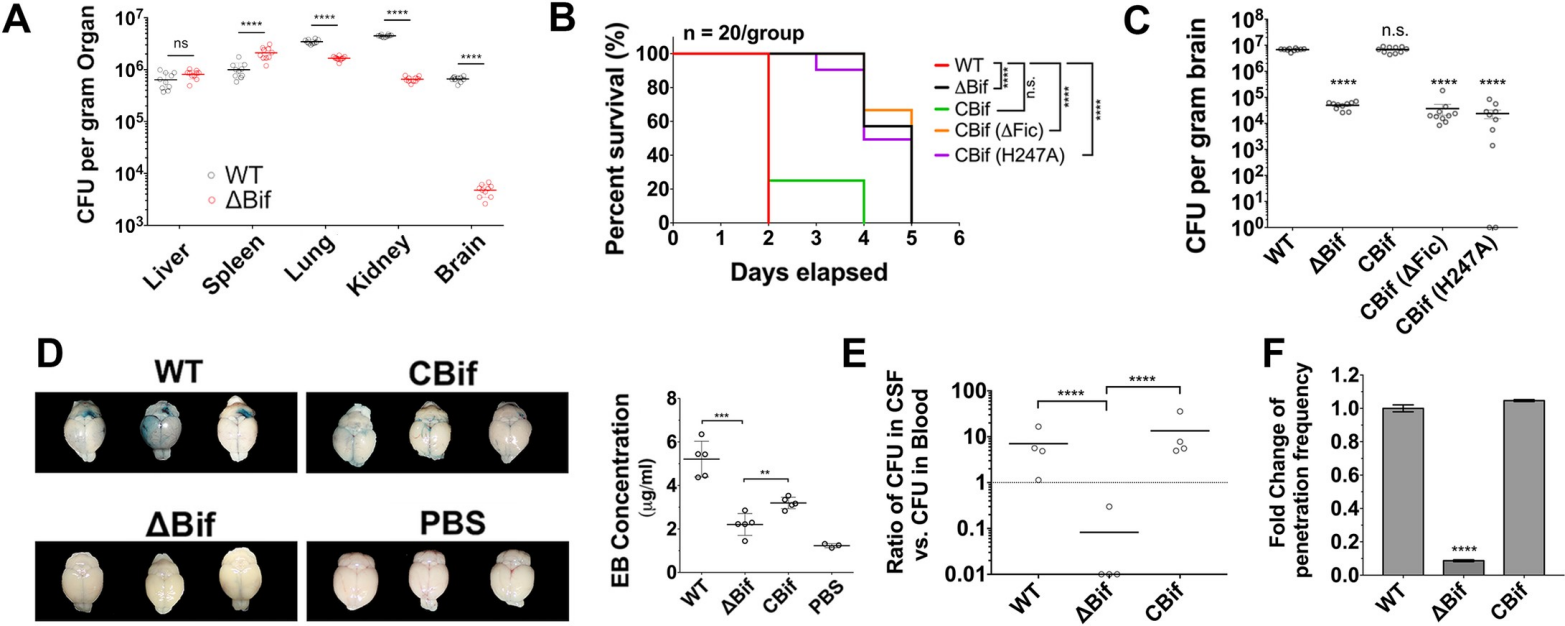
*BifA* enhances SEZ virulence, brain colonization and disrupts the BBB. (A) Recovery of WT or ΔBif CFU from the indicated organs of BALB/c mice 48 hr after i.p. challenge with 1×10^5^ CFU of either SEZ strain (**** indicates p-value <0.0001 with *t* test). (B) Survival curves of BALB/c mice after i.p. challenge with 5×10^5^ CFU WT, ΔBif, or ΔBif complemented with WT BifA (CBif), or BifA deleted for the Fic domain (CBif (ΔFic)), or a BifA H247A substitution mutant (CBif (H247A)). Each group contains 20 mice (**** indicates p value <0.0001 with Log-rank Test vs WT). (C) Burdens of indicated strains in mouse brains 2 days post i.p. challenge with 5×10^5^ CFU, 10 mice/group (**** indicates p-value <0.0001 with *t* test). (D) Evans Blue (EB) permeability in brains of mice infected with the indicated strains. Mice were inoculated with EB 18 hours after they were i.v. inoculated with 5×10^6^ CFU of WT, ΔBif or CBif. Six hours later, brains were dissected and EB was extracted and quantified (** indicates p-value <0.01 and ***indicates p-value <0.001with *t* test). (E) Ratio of bacterial burdens in cerebrospinal fluid versus blood 12h post i.v. infection with indicated strains (4 mice/group, **** indicates p-value <0.0001 with *t* test). (F) WT and mutant SEZ traversal of hBMEC grown in transwell chambers. The Y-axis represents the percentage of bacteria that migrated from the upper to the lower chamber in 3 replicates (**** indicates p-value <0.0001 with *t* test).

We previously found that SEZ ATCC35246 contains 2 *purC* homologues, SeseC_00028 and SeseC_01334. The later locus was presumably acquired by horizontal transfer because its G+C content (34.86%) differs from the chromosomal G+C content (41.65%). Notably, although SeseC_01334 bears some similarity to SeseC_00028, it also features an additional C-terminal Fic domain (S1 Fig), which in several other bacteria has been linked to pathogenicity [12], and an N-terminal RhuM domain that SeseC_00028 lacks. To investigate if SeseC_01334 (here renamed *bifA*, for brain invasion factor A) is required for SEZ ATCC35246 virulence, we generated a *bifA* deletion mutant strain (ΔBif) as well as a complemented strain (CBif), in which BifA was expressed from a plasmid in the ΔBif background. Using an established murine model of SEZ infection [13], mice were inoculated via intraperitoneal (i.p.) injection with WT or ΔBif strains. There was ~100× more WT colony forming units (CFU) than ΔBif CFU recovered from the brains of infected mice (Fig 1A). In contrast, there were less marked differences in numbers of WT and ΔBif CFU recovered from the lung and kidney and in the liver and spleen, the number of ΔBif CFU recovered tended to exceed those of the WT (Fig 1A). Thus, BifA may be particularly important for SEZ colonization of the brain. Furthermore, all WT-challenged mice died by 2 days post-infection (dpi), whereas mice challenged with ΔBif survived until 5 dpi (Fig 1B). Complementation of BifA in the ΔBif mutant restored its lethality to WT levels (CBif, Fig 1B) as well its capacity to colonize the brain (Fig 1C). Despite the differences in the virulence of the WT and ΔBif strains, they had very similar in vitro growth curves (S2A Fig), suggesting that an intrinsic growth defect is not the explanation for the in vivo attenuation of the mutant. Together, these observations show that BifA promotes SEZ’s lethality and its capacity to enter into and/or proliferate in the brain.

For SEZ to colonize the brain it must traverse the BBB. We used an Evans Blue (EB) dye permeability assay [13] to assess the integrity of the BBB in mice inoculated with SEZ. EB was administered to mice 18 hours post infection (hpi) with WT, ΔBif or CBif and then the brains were dissected 2 hours later (Fig 1D). The brains of mice infected with WT SEZ had significantly greater amounts of detectable EB than the brains of mice infected with ΔBif (Fig 1D); *bifA* complementation partially restored the capacity of ΔBif to disrupt the BBB (Fig 1D). Thus, the marked defect of the ΔBif strain to colonize the brain may, at least in part, be explained by the reduced capacity of this strain to penetrate the BBB. Consistent with this hypothesis, we found that there was a much lower ratio of CFU recovered from the CSF vs the blood 12 hour after infection with ΔBif vs the WT strain (Fig 1E), even though there were very similar numbers of WT and ΔBif organisms recovered from blood at this point (S2B Fig). The absence of *bifA* appears to account for the reduced capacity of ΔBif to access the CSF, since this defect was not observed in the complemented strain (Fig 1E). Furthermore, the WT and ΔBif strains had indistinguishable capacities to proliferate in blood (S2B Fig). One consequence of BifA’s apparent capacity to promote SEZ disruption of the BBB may be the severe cerebral hemorrhage that was observed in the brains of animals infected with WT and CBif, but not in those infected with ΔBif (S3 Fig). Moreover, using a transwell assay, we found that WT and CBif had a greater capacity to traverse human brain microvascular endothelial cell (hBMEC) monolayers than ΔBif (Fig 1F). Together, these observations suggest that BifA promotes SEZ virulence and brain pathology by enabling the pathogen to transit the BBB.

### BifA’s Fic domain enables the protein to enter host cells and promotes SEZ virulence

**Fig 2:**
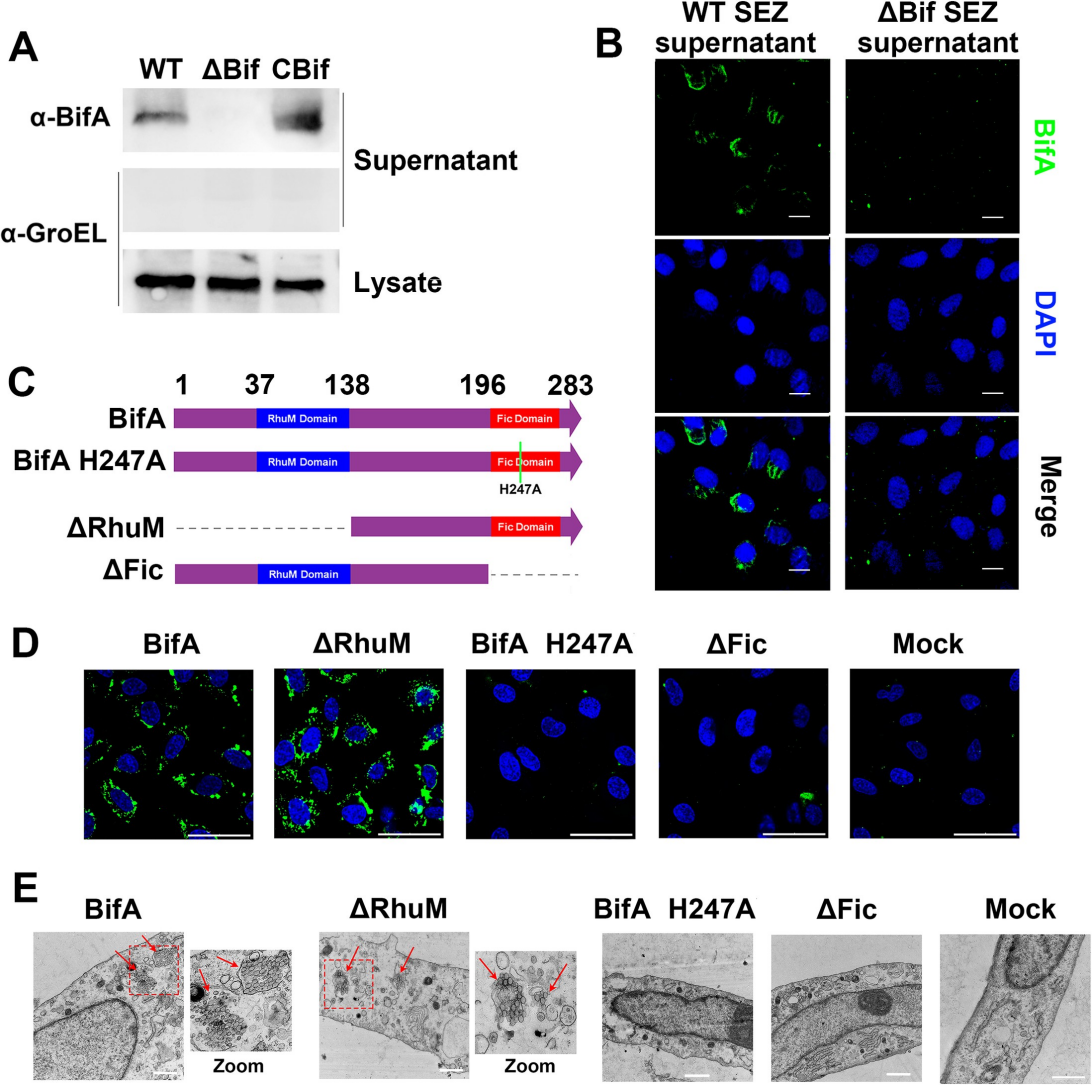
BifA is released into culture supernatants and enters into host cells. **(A)** Detection of BifA in culture supernatants by western blot. Immunoblots were performed with anti-BifA polyclonal antibody. Immunoblots of supernatants with antibody against GroEL, a cytosolic protein, were also performed to check for cytosolic contamination of supernatants and of bacterial lysates as a loading control. **(B)** Immunofluorescence microscopy of hBMECs incubated with supernatants from WT or ΔBif SEZ. BifA was detected by immunostaining with anti-BifA antibody (green) and DAPI was used as a counterstain for nuclei (blue) (scale bar = 10 μm). **(C)** Diagram of wild type and variant BifA proteins purified for experiments in (**D**) and (**E**). **(D)** Immunofluorescence microscopy of hBMECs that had been incubated with indicated BifA variants. BifA was detected by immunostaining (green) and DAPI was used as a counterstain for nuclei (blue). Images represent merged fluorescence channels (scale bar = 50 μm). **(E)** Transmission EM of hBMECs that had been incubated with latex beads coated with indicated BifA variants. For mock, cells were incubated with uncoated latex beads. Dashed boxes indicate areas of higher magnification (shown on right for BifA and ΔRhuM). Arrows indicate internalized latex beads (scale bar = 1 μm).

Prediction of protein structure using the THHMM server suggested that BifA lacks transmembrane helical domains and is likely a hydrophilic protein. We found that BifA could be detected in SEZ culture supernatants (Fig 2A), raising the possibility that it might directly interact with host cells to modulate BBB integrity. Consistent with this idea, we found that BifA could be detected as cytoplasmic foci inside cultured hBMEC cells after exposure to supernatant derived from WT but not ΔBif SEZ (Fig 2B).

The full-length and N- and C-terminal portions of BifA (ΔFic and ΔRhuM, respectively) were purified along with a BifA mutant containing an H247A substitution in the Fic domain (Fig 2C). This mutation was shown to ablate the catalytic activity of other Fic domain containing proteins [14]. Purified full length BifA was taken up into cultured hBMEC cells where it was detected as cytoplasmic foci by immunofluorescence microscopy (Fig 2D). Notably, the concentration of BifA found in the culture supernatants (~18ug/ml, S4 Fig) used above, were similar to the final concentration of purified BifA used to detect BifA entry into hBMEC cells in Fig 2D and to modulate monolayer permeability in experiments described below. Neither ΔFic nor BifA H247A were detected inside the hBMEC cells, whereas the intracellular amount and distribution of the ΔRhuM BifA variant was similar to full length BifA. Thus, the activity of BifA’s Fic domain appears required for the protein to enter host cells, but its RhuM domain is dispensable for this function.

We also tested whether BifA was sufficient to enable latex beads coated with the protein to enter hBMEC cells. Transmission electron microscopy revealed that beads coated with full length BifA or the ΔRhuM truncated variant enabled latex bead internalization (Fig 2E). Beads coated with ΔFic or H247A BifA were not internalized into cells any more than uncoated beads. Together, these observations indicate that BifA can mediate its own entry into hBMEC cells, and that entry appears dependent on a functional Fic domain.

To test whether a functional Fic domain was important for SEZ virulence in vivo, we inoculated mice with the ΔBif strain complemented with BifA lacking the Fic domain (CBif (ΔFic)) or the H247A allele (CBif (H247A)). These strains were similarly attenuated as ΔBif in lethality (Fig 1B) and brain colonization (Fig 1C). These observations strongly suggest that BifA’s Fic domain is required for robust SEZ virulence.

### BifA disrupts the barrier integrity of hBMEC monolayers

**Fig 3:**
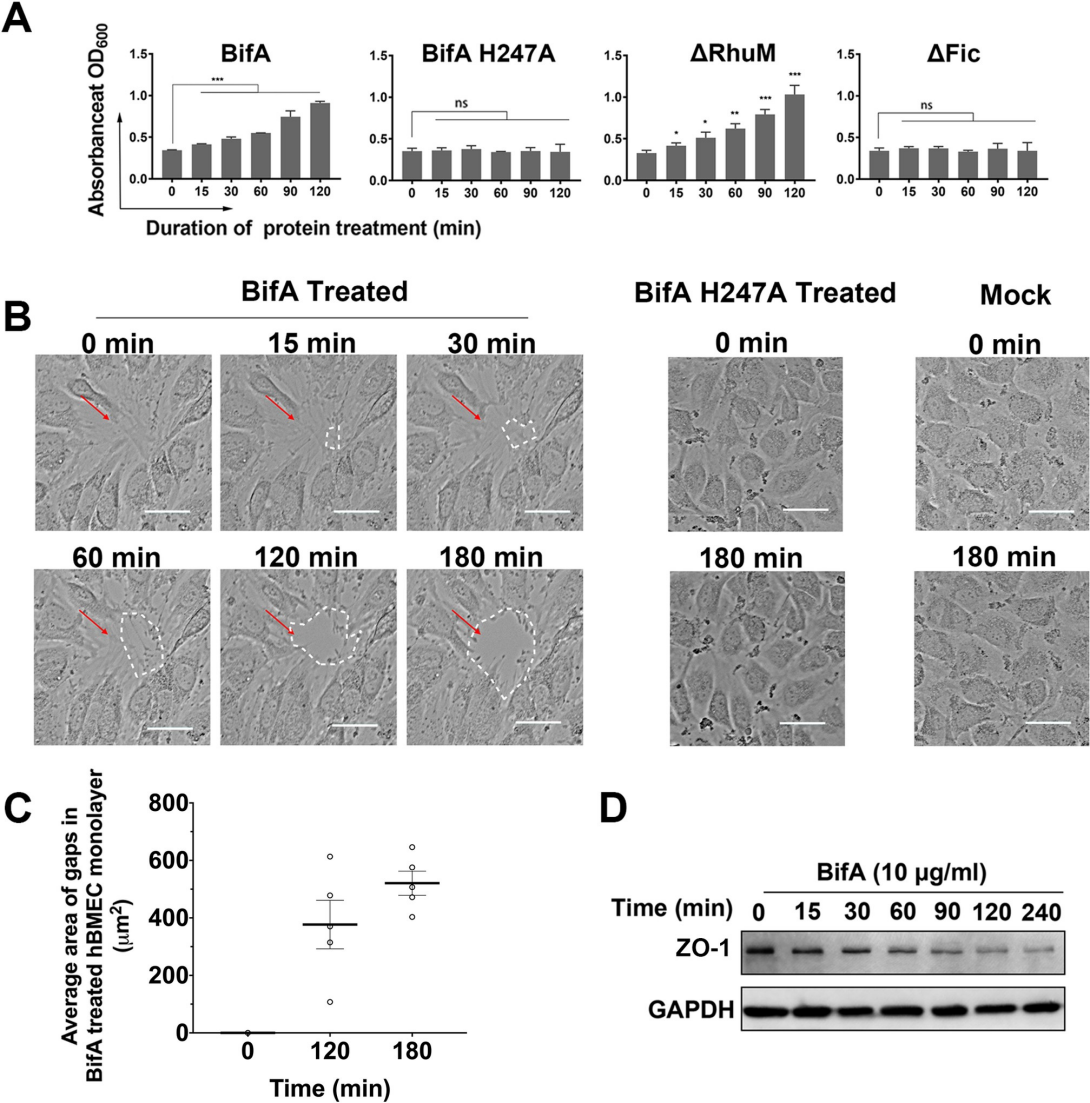
BifA disrupts hBMEC monolayer barrier function. **(A)** Transwell-grown hBMECs were treated with purified BifA or its variants and assessed for barrier integrity with an Evans Blue dye penetration assay (quantitated by absorbance at OD_600_) (results are from 3 experiments, * indicates p-value <0.05, ** indicates p-value <0.01, *** indicates p-value <0.001 with *t* test). **(B)** Time-lapse microscopy of hBMEC monolayers following addition of purified BifA, BifA H247A, or mock treatment with DMEM. Scale bar = 50 μm. For the movies, see supplemental video (S1–S6 Movies). **(C)** Quantitation of gap size in S2 Movie (using Image J) detected in monolayers 2 and 3 hours after treatment with BifA. **(D)** Immunoblot detection of ZO-1 in lysates of hBMEC after BifA treatment. GAPDH was used as reference protein.

Since BifA appears to promote SEZ’s capacity to transit the BBB, we tested whether BifA treatment altered the barrier integrity of hBMEC monolayers. Using penetration of EB as a gauge of barrier disruption [15], addition of either full-length BifA or the ΔRhuM truncation variant to hBMEC monolayers resulted in time-dependent increases in barrier permeability, which became apparent as early as 15 minutes after addition of BifA or ΔRhuM (Fig 3A). In contrast, addition of the H247A BifA mutant or the ΔFic truncation variant to the transwells did not alter the monolayers’ barrier function.

Live microscopy of monolayers was carried out to monitor the effects of BifA and BifA H247A treatment on monolayer integrity (Fig 3B and S1–S6 Movies). In some parts of the BifA-treated monolayers, the hBMEC membranes between adjacent cells appeared to retract by ~15–30 min after addition of the protein and frank gaps in the monolayer, which widened through time, became evident by ~120 min after treatment (Fig 3B from S1 Movie and Fig 3C from S2 Movie). In contrast, addition of H247A BifA to monolayers did not result in detectable morphologic changes in the hBMEC cells compared to the untreated monolayer (mock) over a 3 hours period of observation (Fig 3B, S3–S6 Movies). Additional studies to elucidate the molecular mechanism(s) by which BifA disrupts the integrity of hBMEC monolayers are required. However, interruption of tight junctions could contribute to the permeabilization of the monolayers, since we found that cellular levels of the tight junction protein, zona occludens-1 (ZO-1), decreased after addition of BifA (Fig 3D, S5 Fig).

### BifA binds to the moesin ERMAD domain

**Fig 4:**
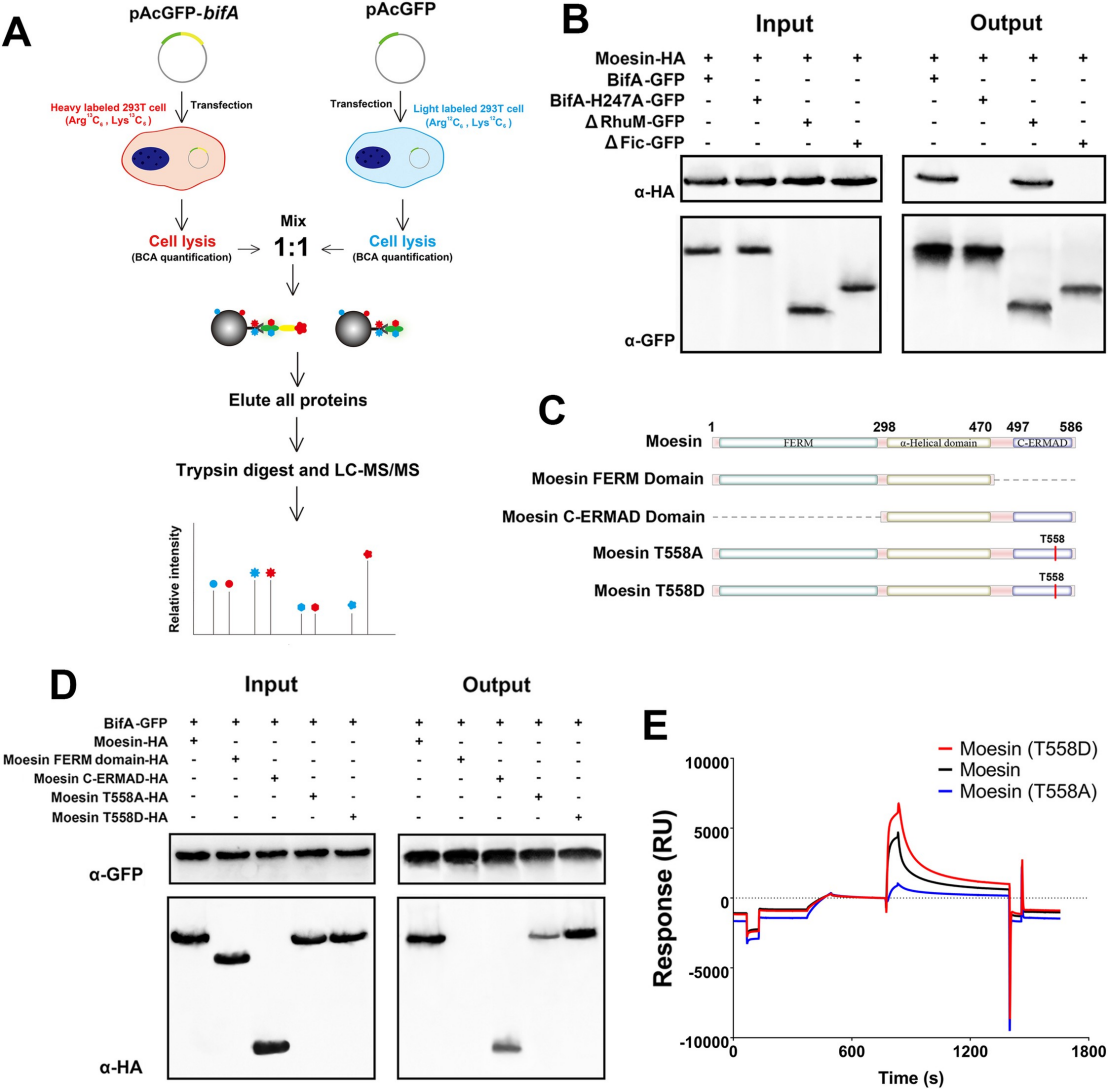
BifA interacts with moesin. **(A)** Schematic of proteomic strategy for identifying BifA binding partner(s). 293T cells were transfected with plasmids encoding GFP-tagged BifA or GFP alone, differentially labelled with heavy isotope (Arg ^13^C_6_, Lys ^13^C_6_) or light isotope (Arg ^12^C_6_, Lys ^12^C_6_), and GFP or GFP-BifA binding partners were compared by SILAC (see methods for details). **(B)** Co-immunoprecipitation of HA-tagged and GFP tagged BifA or its variants. Inputs are immunoblots of moesin and BifA in cell lysates prior to co-IP. Outputs are immunoblots of moesin and BifA in eluates after BifA immunoprecipitation. **(C)** Schematic of moesin variants for experiments in D and E. **(D)** Co-immunoprecipitation of GFP-tagged BifA and HA-tagged moesin variants, immunoblotted as in (B). **(E)** Surface plasmon resonance of the moesin-BifA interaction. The Y-axis shows response units (RU), where 1 RU is equivalent to a change in surface protein concentration of 1 pg/mm^2^.

We used a SILAC-based comparative ‘pull-down’ approach to identify BifA binding partners. For these studies, BifA-GFP was expressed in HEK293T cells and the proteins that precipitated along with BifA were identified by mass spectrometry (Fig 4A). One of the top hits among the 19 candidate BifA-interacting protein identified (S1 Table) was an ERM family protein, which was enriched ~1.8-fold in the BifA-GFP vs the GFP pull-down. ERM family proteins include Ezrin, Radixin, and Moesin, which function in endothelial cells as well in other cell types as critical regulators of the actin cytoskeleton [16]. These proteins are capable of binding to integral membrane proteins through their N-terminal FERM domains and filamentous actin through their C-terminal Ezrin Radixin Moesin association domain (ERMAD). By virtue of these dual binding capacities, ERM proteins regulate actin polymerization at the cell cortex, where they provide a critical link between the cell membrane and cytoskeletal components [17]. Since the dominant ERM family protein in hBMEC is moesin [16], we focused subsequent studies on BifA’s potential interaction with moesin.

To confirm that BifA interacts with moesin in hBMEC cells, co-immunoprecipitation (co-IP) experiments were performed with lysates from hBMEC expressing HA-tagged moesin and several BifA variants. The epitope-tagged moesin co-IPed with full-length BifA and the ΔRhuM truncation variant, but not with the H247A BifA or ΔFic variants (Fig 4B). Thus, BifA’s interaction with moesin in hBMEC cells appears to depend on its Fic domain. Similar co-IP experiments were carried out using cells transfected with tagged variants of moesin (Fig 4C), to determine which of the moesin domains is required for BifA interaction. BifA co-IPed with a moesin variant lacking the FERM domain but not with a variant lacking the ERMAD domain (Fig 4D), suggesting that the FERM domain is dispensable for the BifA-moesin interaction. In addition, we found that purified BifA could interact with moesin in hBMEC lysates, while BifA H247A could not (S6 Fig).

Moesin’s ERMAD domain includes a highly conserved threonine residue (T558) that is phosphorylated during activation [17]. Substitution mutants in the moesin T558 residue that are predicted to be phosphoablative (T558A) or phosphomimetic (T558D) were generated to begin to address whether T558 phosphorylation modulates BifA-moesin interaction. Interestingly, BifA precipated less moesin T558A than WT moesin or moesin T558D, which precipitated with BifA at least as well as WT moesin (Fig 4D), suggesting that BifA’s interaction with moesin is enhanced by phosphorylation of moesin T558.

Surface plasmon resonance analyses with purified BifA and moesin proteins were performed to further characterize BifA’s interaction with moesin. These studies demonstrated that BifA could bind moesin in isolation from other proteins, and thereby indicate the interaction is direct (Fig 4E). Consistent with previous results, minimal binding of the H247A or ΔFic BifA variants to moesin was detected with this assay (S7A Fig). Additionally, the binding affinity of BifA for the moesin T558A mutant was less (T558A, *K*_*D*_ = 7.366×10^−7^ M) than that of T558D (*K*_*D*_ = 1.078×10^−8^ M), which had an even higher affinity than the wild-type protein (Fig 4E and S7B Fig). Collectively, these observations demonstrate a direct interaction between BifA and moesin that is dependent on their respective Fic and ERMAD domains and that is likely enhanced by activation (T558 phosphorylation) of moesin.

### BifA promotes moesin phosphorylation and RhoA-GTP formation

**Fig 5:**
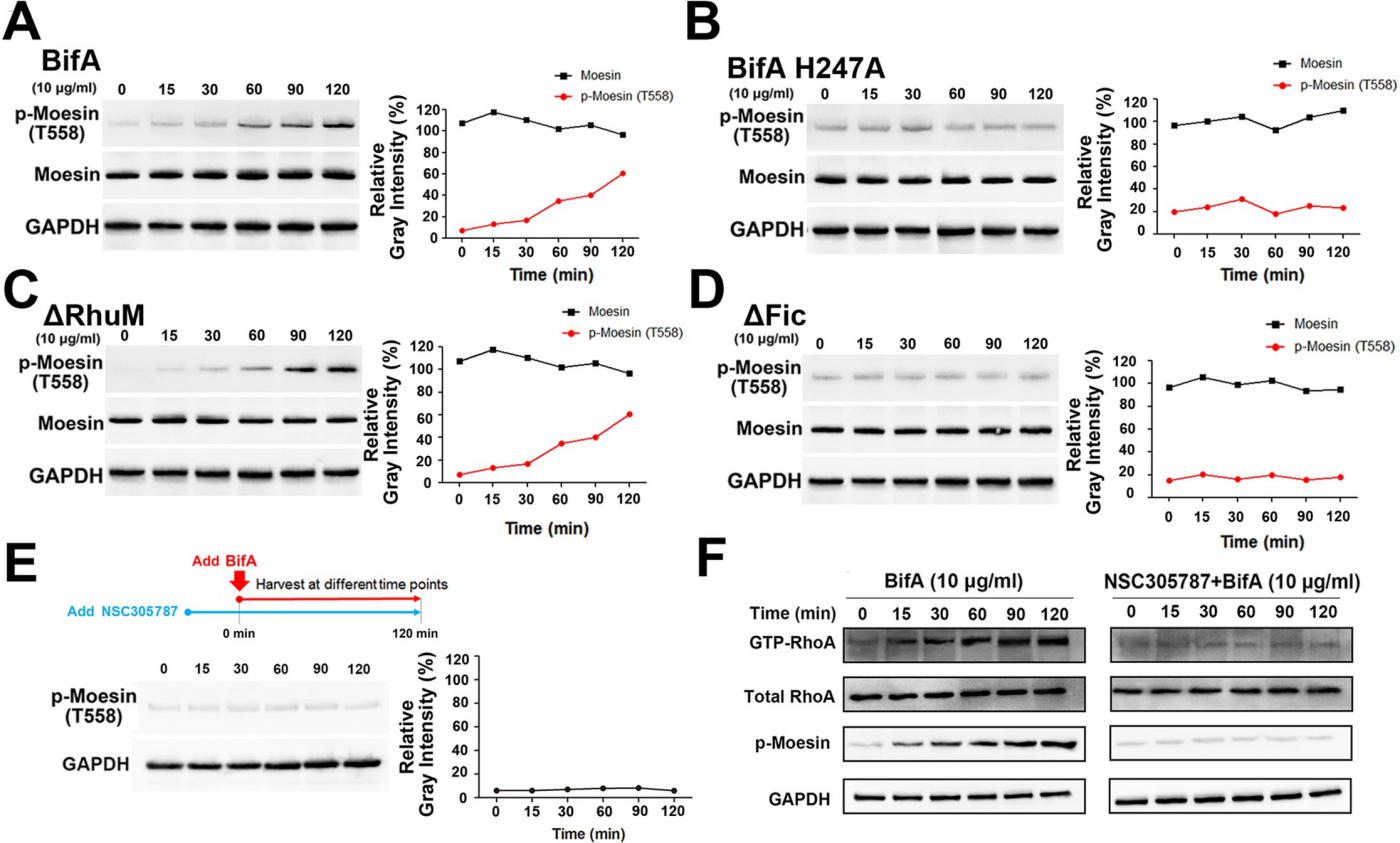
BifA leads to moesin T558 phosphorylation. **(A-D)** Western blot detection of moesin phosphorylation upon BifA treatment. Human BMEC cells were incubated with different BifA variants for the indicated times and lysates were probed with appropriate antibody. GAPDH is a loading control. The graphs on the right panel show the quantitation of the moesin and phospho-moesin bands by ImageJ, normalized with GAPDH. **(E)** Effect of PKC inhibition upon BifA induction of moesin phosphorylation. Cells were pre-treated with PKC inhibitor NSC305787 for 30 min before BifA treatment, then analyzed by immunoblot with phospho-moesin antibody. **(F)** BifA treatment leads to formation of GTP-RhoA. Western blots were performed on lysates of cells treated with BifA alone, or BifA in the presence of NSC305787. Total RhoA and rhotekin protein precipitated GTP-RhoA was detected by anti-RhoA antibody. GAPDH was the loading control.

Since several Fic domain-containing bacterial toxins are reported to lead to the phosphorylation of their respective target proteins [18], we tested whether BifA promotes moesin phosphoryation. We monitored moesin T588 phosphorylation following addition of different BifA variants to hBMEC cells by immunoblotting with an antibody that recognizes phosphorylated moesin T588 (p-Moesin). Addition of either full length BifA or the BifA ΔRhuM variant to cells led to moesin T588 phosphorylation in a time-dependent fashion but did not alter total cellular moesin levels (Fig 5A and 5C). In contrast, no changes in moesin phosphorylation or levels were detected when the H247A BifA or ΔFic variants were added to cells (Fig 5B and 5D). Similar results were obtained from immunoblots of lysates electrophoresed with Phos Binding Reagent Acrylamide, which alters the electrophoretic mobility of phosphorylated proteins (S8 Fig). These observations indicate that treatment of hBMECs with internalizable and moesin-binding variants of BifA promotes moesin phosphorylation.

ERM family proteins are phosphorylated by protein kinase C (PKC) [19]. We used NSC305787, a small molecule inhibitor of PKC phosphorylation of ERM family proteins [20], to investigate whether BifA-induced phosphorylation of moesin was dependent on PKC. When hBMEC cells were pre-treated with NSC305787 for 30 minutes before addition BifA, there was no induction of moesin phosphorylation (Fig 5E), consistent with the idea that BifA induction of moesin phosphorylation depends on PKC.

Moesin phosphorylation leads to activation of small G proteins, such as RhoA and Rac1 [21], that regulate actin cytoskeletal and membrane protrusion dynamics [22], phenotypes that could be pertinent to BifA-induced changes in brain endothelial cells and BBB permeability. RhoA and Rac1 activation are controlled by their conversion from GDP- to GTP-bound states [23], and we used immunoblots to monitor GTP-RhoA and GTP-Rac1 levels in BifA-treated cells. There was a time-dependent increase in GTP-RhoA that was associated with moesin phosphorylation in BifA-treated cells (Fig 5F). GTP-Rac1 levels were also increased during BifA treatment (S9 Fig). When BifA-induced moesin phosphorylation was blocked with NSC305787, GTP-RhoA formation was abrogated (Fig 5F), consistent with the idea that RhoA activation by BifA is dependent on moesin phosphorylation.

### Inhibition of moesin activation and moesin knockdown block BifA-mediated increases in hBMEC permeability

**Fig 6:**
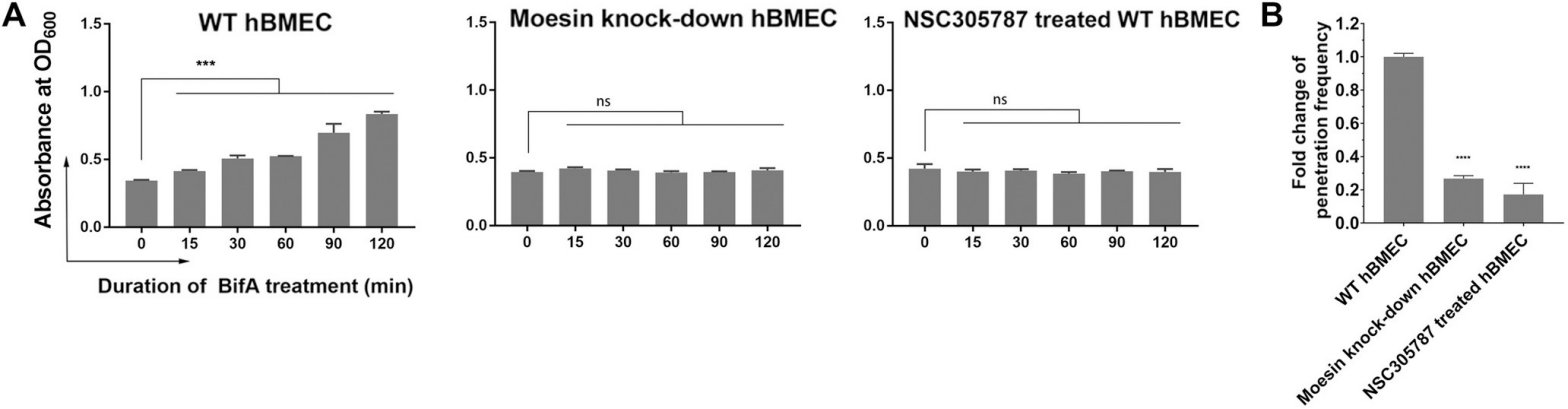
Inhibition of moesin phosphorylation or moesin knockdown block BifA disruption of monolayer barrier functions. **(A)** NSC305787 treatment and moesin KD prevent BifA-induced increase in monolayer permeability. Transwell-grown hBMEC monolayers were assayed for barrier integrity by Evans Blue dye (data represent 3 replicates /condition, *** indicates p-value <0.001 with *t* test). **(B)** NSC305787 treatment or moesin KD inhibit SEZ penetration of hBMEC monolayers. Transwell-grown hBMEC monolayers were infected with SEZ by inoculation into the upper chamber, and the proportion of bacteria in the lower chamber were quantitated by CFU plating (data represent 3 replicates /condition, **** indicates p-value <0.0001 with *t* test).

We next tested whether moesin phosphorylation was required for BifA-mediated barrier disruption. Monolayers pre-treated with NSC305787 did not exhibit increased permeability after addition of BifA (Fig 6A). Similarly, NSC305787 pre-treatment led to marked reduction in SEZ translocation across hBMEC monolayers (Fig 6B). Furthermore, we used siRNA to knockdown (KD) moesin in hBMECs (S10 Fig) to further investigate the requirement of moesin for BifA action. By itself, moesin KD did not alter the barrier function of the hBMEC monolayer, as these cells remained impermeant to Evans Blue dye (Fig 6A). However, the moesin KD cells exhibited significantly less permeabilization after BifA treatment, in marked contrast to control hBMEC monolayers treated with BifA (Fig 6A). Moreover, there was a marked reduction in the ability of SEZ to translocate across the moesin KD monolayer vs the WT monolayer, phenocopying the effects of NSC305787 (Fig 6B). Since NSC305787 inhibits all ERM family protein phosphorylation, the similarity of the phenotypes observed in the NSC305787 treated and moesin KD cells supports that idea that moesin is the dominant ERM protein in hBMECs. Thus, both blockade of moesin phosphorylation and moesin depletion are sufficient to protect cells from BifA-dependent bacterial translocation across the hBMEC monolayer. Collectively, these observations suggest that BifA disruption of hBMEC monolayer barrier integrity relies on moesin-dependent signaling pathways.

## Discussion

We found that the virulence of SEZ ATCC35246 depends on BifA, a Fic domain-containing protein encoded in a pathogenicity island. Deletion of *bifA* reduced its lethality in mice as well its capacity to disrupt the BBB, to colonize the brain and to traverse hBMEC monolayers in tissue culture. BifA bound to moesin, a host protein that regulates cytoskeletal processes. Inactivation of BifA’s Fic domain eliminated its capacity to enter hBMEC monolayers, increase monolayer permeability, and to bind to moesin. BifA activation of moesin appears to be critical for BifA’s modification of monolayer permeability, since either moesin knock down or pharmacologic inhibition of moesin activation abolished BifA-mediated increases in hBMEC permeability and SEZ penetration of a hBMEC monolayer. Collectively, our findings suggest that by usurping moesin-dependent signaling, BifA enables SEZ to efficiently penetrate the BBB.

Our observation that addition of BifA to hBMEC monolayers induced formation of gaps and increased monolayer permeability is consistent with the idea that BifA enables SEZ penetration of the BBB by disrupting the integrity of the brain endothelial monolayer, a critical constituent of the BBB. Concordant with this model, BifA’s action appears dependent on the associated phosphorylation of the ERM protein moesin, which is known to have diverse consequences that include activation of signaling pathways involved in cell adhesion, migration and invasion [21, 24–26]. In particular, we found that moesin phosphorylation following BifA treatment was linked to formation of RhoA-GTP, the active form of this small G protein known to regulate multiple cytoskeletal processes [27, 28]. Formation of RhoA-GTP is likely a consequence of moesin phosphorylation, since blockade of moesin phosphorylation with NSC305787 inhibited the generation of RhoA-GTP (Fig 5F). Notably, activation of RhoA has been shown to promote the dissolution of tight junctions, which serve to limit paracellular permeability between endothelial cells [29]. Loosening of tight junctions and additional factors (e.g. adherens junctions) that increase the adherence of adjacent cells in the brain endothelium could open a paracellular route for SEZ movement across the BBB (S11 Fig).

Additional pathogens manipulate RhoA signaling to reduce the integrity of the BBB. For example, *E*. *coli* K1’s CNF toxin’s modulation of RhoA activity is thought to be important for this common agent of neonatal meningitis to cross the BBB [5]; however, in this case, RhoA activation is thought to enable this pathogen to traverse the BBB via a transcellular route. RhoA activation by the brain-invasive fungal pathogen *Cryptococcus neoformans* also facilitates its traversal of the BBB [30]. Additional Fic domain-containing proteins are known to catalyze post-translational modification of Rho family proteins (e.g. AMPylation of Rho proteins by *Vibrio parahaemolyticus* VopS [12]), but to our knowledge, other Fic toxins have not been reported to modify BBB function.

Both the mechanisms of BifA release from SEZ and uptake into eukaryotic cells require elucidation. In contrast to several Fic domain-containing virulence factors described in other pathogens (e.g. VopS), BifA delivery into the eukaryotic cytosol does not rely on a bacterial type III or type IV secretion system. In this regard, BifA functions as a traditional bacterial toxin, mediating its own uptake into host cells. Similar to BifA, IbpA, a Fic-domain containing protein from the cattle pathogen *Histophilus somni* doesn’t require additional bacterial factors for uptake into bovine cells, where it AMPylates Rho family proteins [12]. It will be particularly interesting to determine whether the receptor(s) and pathways that mediate BifA uptake into host cells modulate its downstream function(s).

The characterized Fic domain-containing proteins produced by other pathogens catalyze post-translational modifications of target proteins upon entry into the eukaryotic cell cytosol [12]. Fic domain-containing proteins can directly phosphorylate their targets; e.g., Doc phosphorylates its target EF-Tu [31]. However, although we found that BifA directly binds to moesin (Fig 4) and that BifA treatment of endothelial cells resulted in elevated levels of phosphorylated moesin (Fig 5), we did not directly demonstrate that BifA phosphorylates moesin. The observation that the PKC kinase inhibitor NSC305787 blocked the induction of moesin phosphorylation in cells treated with purified BifA suggests that BifA may not directly phosphorylate moesin, but could instead promote its phosphorylation indirectly. For example, BifA could enhance the activity of a host kinase, akin to the action of the *Pseudomonas syringae* Fic-like T3SS effector AvrB, which leads to the phosphorylation of the plant immune regulator RIN4 by promoting the activity of the endogenous kinase MPK4 [32]. Alternatively, our observation that BifA binds to the phosphorylated form of moesin with greater affinity than to the non-phosphorylated form (Fig 5), raises the possibility that BifA stabilizes phospho-moesin, leading to its accumulation.

Interestingly, despite a high degree of overall conservation among sequenced SEZ isolates, *bifA* homologues are not found in other SEZ genomes. BifA is encoded in a SEZ ATCC35246 pathogenicity island, suggesting that this critical SEZ Fic-domain containing virulence factor was likely acquired via horizontal gene transfer, and thus that lateral gene exchange was a key step in the evolution of SEZ ATCC35246 as a pathogen. Acquisition of *bifA* alone may be sufficient to enhance BBB penetration by related organisms. BifA homologues are not present in other well-characterized meningeal pathogens, e.g. Group B streptococci, consistent with the idea that different CNS pathogens rely on distinct factors to traverse the BBB [5]. However, BifA homologues with intact Fic domains are present in a variety of other Gram-positive as well as Gram-negative organisms, many of which are usually thought of GI tract commensals, suggesting that BifA-like proteins may carry out functions beyond diminishing the integrity of the BBB. Finally, BifA’s capacity to increase BBB permeability may have medical applications in delivery of drugs and other agents to the brain.

## Materials and methods

### Bacterial and eukaryotic cell culture

*Streptococcus equi* subsp. *zooepidemicus* ATCC35246 (SEZ) was isolated from a dead pig in Sichuan Province, China. SAICAR gene SeseC_01334 (Genbank) was re-named *bifA*. A *bifA* deletion mutant and complemented strain were constructed using pSET4s, a *Streptococcus*-*E*. *coli* temperature sensitive suicide shuttle vector and expression plasmid pSET2 respectively (SI Appendix) [33]. SEZ was cultured in Todd Hewitt Broth (THB) (Becton, Franklin Lakes, NJ, USA) at 37°C. Human brain microvascular endothelial cells (hBMECs) were purchased from ScienCell Research Laboratories (Catalog #1000). HEK293T cells were purchased from American Type Culture Collection (ATCC number CRL-3216). Cells were cultured in DMEM (Gibco, Grand Island, NY, USA) with 10% fetal bovine serum (FBS) (Gibco, Grand Island, NY, USA) in a 37°C incubator containing 5% CO_2_.

### Plasmid construction

The vectors pAcGFP-C and pCMV-C-HA were used for the respective expression of BifA and moesin in eukaryotic cells respectively. *E*. *coli* BL21 (DE3) plysS was used to express recombinant BifA and its variants with the pCold-SUMO vector; *E*. *coli* Rosetta (DE3) was used to express moesin and its variant proteins with pGEX-6p-1. The *bifA* gene was PCR amplified from SEZ genomic DNA and subcloned into the pAcGFP-C vector. The moesin cDNA was PCR amplified from human cDNA and subcloned into the pCMV-C-HA vector. For expression of His- or GST-tagged proteins, *bifA* was subcloned into pCold-SUMO vectors and moesin was subcloned into pGEX-6p-1. The mutations in *bifA* and moesin were constructed by PCR mutagenesis using the ClonExpress II One Step Cloning Kit (Vazyme Biotech Co., China). The plasmid constructs were verified by Sanger sequencing. *E*. *coli* DH5α was used for propagation of plasmids. All plasmid information and primers are listed in S2 Table.

### ΔBif and complementary strain construction

An allele exchange vector for deletion of bifA was created by PCR amplification of fragments upstream and downstream of the *bifA* gene with primers of *bifA*-up-fwd/*bifA*-up-rev and *bifA*-dwn-fwd/*bifA*-dwn-rev, using SEZ ATCC35246 genome as template. The upstream and downstream PCR products were mixed 1:1, and primer pair *bifA*-up-fwd/*bifA*-dwn-rev were subjected to fusion PCR amplification. The fusion fragment was purified, digested with appropriate endonucleases, and then cloned into the same sites of the temperature-sensitive *S*. *suis*-*E*. *coli* shuttle vector pSET4s [33]. Plasmids were electroporated into *SEZ* (Bio-Rad, Gene Pulser Xcell, Voltage: 2300V, Capacitance: 25μF, Resistance: 200Ω, Cuvette: 1mm) and mutant isolation was carried out as described [34]. The *bifA* gene was amplified using the primer pair *bifA*-pSET2-fwd and *bifA*-pSET2-rev using SEZ genomic DNA as the template, and then inserted into the pSET-2 plasmid. This plasmid was used to complement the bifA deletion in the ΔBif strain. Templates containing mutant versions of bifA were amplified from the expression vectors used above and subcloned into pSET-2. The inserts in all plasmids were confirmed by Sanger sequencing.

### Ethics statement and animal experiments

All animal experiments were performed with protocols approved by the College of Veterinary Medicine of Nanjing Agricultural University for Research Protection Standing Committee on Animals in accordance with Science and Technology Agency of Jiangsu Province guidelines (SYXK2017-007). All efforts were made to minimize animal suffering.

Four-week-old female BALB/c mice, purchased from the Comparative Medicine Center of Yangzhou University, were used for all animal work. In bacterial load determination of different organs in Fig 1A, mice (10/group) were i.p. challenged with 1×10^5^ CFU of WT or mutant SEZ. In the mortality experiments shown in Fig 1B, mice (20/group) were i.p. challenged with 5×10^5^ CFU of WT or mutant SEZ. For bacterial load determinations in Fig 1C, mice (10/group) were i.p. challenged with 5×10^5^ CFU of WT or mutant SEZ and CFU counts from the brain were determined 2 days later. For pathology (S3 Fig), brains were harvested and embedded in paraffin and sectioned for hematoxylin and eosin staining, 48 hours after intravenous injection of 1×10^6^ CFU of WT and mutant SEZ.

Evans Blue (EB) leakage was used to assess BBB permeability as described [13]. In these experiments, mice were challenged i.v. with 5×10^6^ CFU of WT or mutant SEZ, and 18 hour later, 100μl of 2% EB was administered i.v. Two hours later, brains were dissected, photographed and then dried at 56°C in aluminum foil for two days. Formamide was used to extract the EB out of the tissue and EB amounts were determined as absorbance at OD_620_.

### Protein purification

To purify His-tagged BifA and its variants, cultures of BL21 (pCold-SUMO-*bifA*) was grown to OD_600_ = 1.0 in Luria-Bertani (LB) broth at 37°C, 180 rpm then induced with IPTG at a final concentration of 1 mM at 16°C, 180 rpm for 24 hours. Bacterial cells were collected by centrifugation. Cells were lysed in the lysis buffer (10mM Tris-HCl, pH 8, containing 100 mM NaCl and 20mM imidazole) by sonication. The cell lysate was centrifuged and the supernatant was used for purification. SUMO tag was digested with SUMO protease (Thermo, Waltham, MA, USA). Primary purification was performed using the Histrap HP column (GE Healthcare, Piscataway, NJ, USA), followed by Superdex 200 10/300 GL gel filtration (GE Healthcare, Piscataway, NJ, USA) using an AKTA Protein Purifier (GE Healthcare, Piscataway, NJ, USA).

To purify non-tagged moesin for SPR, cultures of BL21 (pGEX-6p-1-*msn*) were grown until OD_600_ = 0.8 in LB at 37°C, 180 rpm then with IPTG at a final concentration of 1 mM at 30°C, 180 rpm for 5 hours. Cells were lysed in the lysis buffer (PBS, 140 mM NaCl, 2.7 mM KCl, 10 mM Na_2_HPO_4_, 1.8 mM KH_2_PO_4_, pH 7.3) by sonication. The lysates were used for purification with GSTrap HP 5 ml column (GE Healthcare, Piscataway, NJ, USA) followed by tag digestion with Precission Protease and GSTrap FF 1 ml column (GE Healthcare, Piscataway, NJ, USA), Sephadex 10/300 (GE Healthcare, Piscataway, NJ, USA) was used to final purification in AKTA Protein Purifier (GE Healthcare, Piscataway, NJ, USA).

### In vitro BBB model construction and assay

WT hBMEC or moesin knock-down hBMEC were seeded on the apical side of collagen-coated polytetrafluoroethylene (PTFE) 3 μM pore-size membranes (Corning Incorporated, Corning, NY, USA) for the bacterial penetration assay and 0.4 μM pore-size membranes (Corning Incorporated, Corning, NY, USA) for the barrier integrity assay. Cells were grown for 7 to 10 days to form intact monolayers. Barrier integrity was assessed with 0.4% Evans Blue solution [35].

Bacterial cells (1×10^6^ CFU) were added to the upper chamber of transwells containing hBMEC and incubated at 37°C in 5% CO_2_ for 2 hours. The 100 μl medium from both sides of the transwells was collected and spread on THB agar plates for CFU determination [36]. To examine the effect of BifA on barrier integrity, 10 μg/ml BifA was added to the upper chamber of transwells. Transwell inserts were then transferred to a fresh plate containing Hanks Balanced Salt Solution (HBSS) in the bottom chamber and 50 μl of 0.4% Evans Blue solution in PBS was added to the upper chamber. Transwell inserts were incubated at 37°C in 5% CO_2_ for 40 min, and the permeability was assessed by colorimetric quantification at OD_600_ nm of the bottom chamber as described [37].

### Analysis of bacterial proteins in culture supernatant

SEZ was grown to an OD_600_ = 0.6 in THB media at 37°C with vigorous shaking (180 rpm). Bacteria were diluted 1:100 in DMEM media and grown for 12 h at 37°C with vigorous shaking (180 rpm) and then culture supernatants were isolated by centrifugation. Proteins were precipitated from supernatants with TCA-acetone as described [38, 39].

### Protein binding to latex beads and electron microscopy

Approximately 10^6^ sulfate-modified fluorescent red polystyrene latex beads (0.1 μm mean particle size, Sigma-Aldrich, St. Louis, MO, USA) were suspended in 200 μL of 25 mM 2-(N-morpholino) ethanesulfonic acid (MES) buffer, pH 8.0. Purified BifA or BifA variants were dissolved in 10 μL 25mM phosphate buffer (pH 7.2) and incubated with the suspended beads at 4°C overnight with gentle mixing. We sequentially added 25mM MES buffer (150 μL) every 15 min until the original volume was diluted 200-fold. The coated beads were collected by centrifugation at 3000 × g for 20 min, washed twice in MES buffer and resuspended in DMEM without FBS and then sonicated for dispersal. Dot blot assays were used to confirm protein coating of the beads [40].

For transmission electron microscopy, coated beads were incubated with hBMEC (100:1) for 4h at 37°C. Extracellular beads were removed by washing with PBS and then samples were fixed in 2.5% paraformaldehyde and 0.1% glutaraldehyde in 0.05 M cacodylate buffer, pH 7.3. Then, 0.03% CaCl_2_ was added to the mixture. After fixation, the cells were washed with 0.1 M cacodylate buffer, and pelleted by centrifugation. Low melting point agar was pre-embedded and stained with 1% uranyl acetate in 0.1 M maleate buffer, then dehydrated in ethanol. Ultrathin sections were cut, stained with lead citrate and examined using a JEM 1400-PLUS electron microscope (JEOL, Tokyo, Japan) [41].

### Immunofluorescence

The hBMEC were seeded onto 15 mm Glass Bottom Cell Culture Dishes (Corning, NY, USA) and treated with 10 μg/ml BifA or BifA variants for 2 hours. Cells were then fixed with 4% paraformaldehyde followed by 0.1% Triton X-100 permeabilization buffer and blocked with 5% BSA in PBS-Tween. Mouse polyclonal anti-BifA antibody, Alexa 488-conjugated goat anti-mouse antibody (Jackson Immunoresearch, West Grove, PA, USA), rabbit anti-Moesin antibody (Abcam, Cambridge, MA, USA) and Alexa 594-conjugated goat anti-rabbit antibody (Jackson Immunoresearch, West Grove, PA, USA) were used at 1:2000 in PBS containing 1% BSA. Primary antibodies were incubated for 2 hours and secondary antibody for 1.5 hours at room temperature. 4,6- Diamidino-2-phenylindole‎ (DAPI) was used to detect cell nuclei. Plates were washed three times with Phosphate buffered saline with Tween-20 (PBST) with shaking to wash out unbound antibodies. Images were obtained on a laser scanning confocal microscope (LSCM) (ZEISS, Japan).

### Live-cell imaging

The hBMEC cells were cultured on 6-well Glass Bottom Plates (∅35mm, Cellvis, CA, USA) for 7–9 days until monolayers were confluent. Cells were replenished with DMEM medium, and BifA or BifA variants, at a final concentration of 10 μg/ml, was added to the wells. The plates were cultured in a controlled environmental chamber at 37°C in 5% CO_2_. Time-lapse images were acquired at an interval of 30 s for 300 min through an EC Plan-Neofluar 20×/0.50 M27 lens on an Axiom Observer.Z1/7 microscope, using the Applied Precision motorized stage (Carl Zeiss, Japan). ZEN software was used for image processing.

### Protein interaction screen

Stable isotope labelling of amino acids in cell culture (SILAC) was used to identify BifA interacting host proteins in HEK293T cells. Cells were labeled with heavy isotopes (Arg^13^C_6_, Lys^13^C_6_) or light isotopes (Arg^12^C_6_, Lys^12^C_6_) in Dulbecco’s modified Eagle medium (DMEM) with 10% FBS (Pierce, Rockford, IL, USA) at 37°C in 5% CO_2_ as previously described [42]. The cells were passaged for 6 generations to ensure adequate labeling of proteins. The heavy and light labeled cells were seeded in 10 cm cell culture dishes and transfected with 24 μg of pAcGFP-BifA or pAcGFP using Lipofectamine 2000 (Thermo, Waltham, MA, USA) respectively. Transfected cells were lysed in 500 μl cold Mammalian Protein Extraction Reagent (Thermo, Waltham, MA, USA), containing a protease inhibitor cocktail (Thermo, Waltham, MA, USA) and centrifuged at 14000 g for 10 min at 4°C. Protein concentrations were measured using the BCA Protein Assay Kit (Pierce, Rockford, IL, USA) according to the manufacturer’s directions. We mixed equal quantities of heavy and light lysates and pre-cleared them on Protein G agarose (Santa Cruz, Santa Cruz, CA, USA) with 100 μg mouse IgG (CMCTAG, Milwaukee, WI, USA) for 1h at 4°C with gentle agitation. Pre-cleared lysates were centrifuged and the supernatants transferred to new tubes. Mouse anti-GFP antibody (CMCTAG, Milwaukee, WI, USA) was added to the cold lysates and incubated at 4°C for 1 h, then 40 μl protein G agarose was added and incubated at 4°C on a rotating device overnight. Beads were washed five times with 1 ml of cold PBS. After the final wash, the bound proteins were eluted with 50 μl of elution buffer (50mM Tris-HCl, 1% SDS) and samples were boiled for 5 min. The eluted proteins were digested as previously described [35]. The peptides were separated by reverse-phase liquid chromatography using a nano-LC system (DIONEX Thermo Scientific) and analyzed by tandem mass spectrometry using an LTQ-Orbitrap mass spectrometer (Thermo Scientific) with a nanoelectrospray ion source.

### Co-immunoprecipitation

HEK293T cells were seeded in 10 cm cell culture dishes, which were each transfected with 24 μg of pAcGFP-BifA and pCMV-HA-Moesin, or pAcGFP and pCMV-HA plasmids. In addition, truncated fragments of moesin were obtained by PCR and cloned into pCMV-HA plasmids. The resulting plasmids, pCMV-HA-Moesin FERM domain (1-470aa) and pCMV-HA-Moesin C-ERMAD domain (470-577aa) were co-transfected with pAcGFP-BifA plasmid respectively. Lysates were harvested 48 h later with lysis buffer and cleared by centrifugation as described above. Twenty microliters of lysate was saved for analysis of the expression efficiency and the remainder was immunoprecipitated with Protein G agarose bound to either anti-HA or anti-GFP specific antibody (CMCGAT, Milwaukee, WI, USA). Immunoprecipitated beads were resuspended and boiled for 5 min in 1× Laemmli sample buffer and then used for Western blot analysis.

### Western blot

Boiled cell lysates were subjected to SDS-PAGE, followed by transfer to a PVDF membrane (Roch, Basel, Switzerland) using a semi-dry transfer apparatus (GE Healthcare). Membranes were blocked in 5% non-fat milk powder in TBS containing 0.01% Tween 20 (TBST). Primary antibodies were used and diluted as follows: 1:500 anti-BifA rabbit polyclonal antibody; 1:1000 anti-Moesin (Abcam, Cambridge, MA, USA); 1:1000 anti- phospho T558-Moesin (Abcam, Cambridge, MA, USA); 1:2000 anti-HA (CMCGAT, Milwaukee, WI, USA); 1:2000 anti-GFP (CMCGAT, Milwaukee, WI, USA); 1:2000 Anti-ZO-1 tight junction protein antibody (Abcam, Cambridge, MA, USA) and 1:2000 anti-GAPDH (CMCGAT, Milwaukee, WI, USA). Membranes were incubated with primary antibody diluted in TBST containing 1% BSA overnight at 4°C and then washed for 30 min in TBST. This was followed by incubation with 1:5000 HRP goat anti-rabbit or goat anti-mouse IgG antibody (ABGENT, San Diego, CA, USA). Membranes were washed for 3 × 15 min in TBST before adding ECL reagent (Thermo, Waltham, MA, USA). Chemiluminescence was detected on a ChemiDoc system (Bio-Rad, Hercules, CA, USA).

### Surface plasmon resonance

The direct binding potential of BifA and moesin was analyzed using the Biacore X100 instrument (GE Healthcare). Recombinant His-BifA protein and its variants were separately immobilized onto Biacore NTA sensorchips (GE Healthcare). The recombinant moesin protein, moesin T558A or moesin T558D were injected individually and the binding interactions were recorded. Results were analyzed using the Biacore X100 Evaluation Software (GE Healthcare).

### Detection of moesin phosphorylation and RhoA activation assay

The hBMEC were seeded into 6-well plates (Corning Incorporated, Corning, NY, USA). For serum starvation, once cells reached 70% confluency, the medium was replaced by low serum medium (1% FBS) for 24h, and then replaced with DMEM without FBS and incubated for 16–20 h. After serum starvation, BifA or BifA variants at a concentration of 10 μg/ml in DMEM were added to cells. At different time points (0–120 min), cells were harvested for protein extraction. Cells were lysed with M-PER Mammalian Protein Extraction Reagent (Thermo, Waltham, MA, USA) on ice in the presence of Halt Protease and Phosphatase Inhibitor Cocktail, EDTA-free (100×). In some cases, cells were pretreated with 10 μM of a PKC inhibitor of ERM protein phosphorylation, NSC305787 [20] (MedChemExpress, Monmouth Junction, NJ, USA) for 30 min prior to addition of BifA. Phosphorylation levels of extracted proteins were detected by Western blot with anti-phospho T558-Moesin antibody (Abcam, Cambridge, MA, USA).

Phos Binding Reagent Acrylamide (PBR-A) (APExBIO, TX, USA) was also used to detect moesin phosphorylation. Briefly, extracted proteins were electrophoresed in a 6% acrylamide gel containing 50 mM PBR-A and 10 mM Mn^2+^. After electrophoresis, the gel was soaked in a general transfer buffer containing 10 mmol/L EDTA for a minimum of 10 minutes with gentle agitation, followed by gentle agitation in buffer without EDTA for 10 minutes. The gel was then transferred to PVDF membranes for Western-blotting with a rabbit anti-Moesin antibody (Abcam, Cambridge, MA, USA)

The amounts of GTP-RhoA or GTP-Rac1 in cell lysates were measured by a pull-down method based on specific binding to Rhotekin- RBD coated beads for 1h at 4°C under gentle rotation, followed by western blot with an antibody specific for the GTP-bound form of RhoA or Rac1(RhoA/Rac1/Cdc42 Activation Assay Combo Biochem Kit, Cytoskeleton, Inc., USA). Total RhoA or Rac1 in cell lysates were detected by anti-RhoA and anti-Rac1 antibodies respectively.

### Creation of moesin knock-down hBMEC cell lines

An shRNA targeting the moesin gene was ligated into the pLVX-shRNA1 vector (Clontech, Mountain View, CA, USA). The lentivirus was packaged using commercial reagents (Applied Biological Materials, Nanjing, JS, China). Lentiviral particles (MOI = 1) were added to the hBMEC and seeded in 6-well plates, with media change after 24 h of incubation. After transduction, cellular protein was extracted for Western blot detection, while the RNA was extracted at different time points for Quantitative Real-Time PCR detection. Moesin transcripts levels were determined using the ABI Prism 7300 and Sequence Detection System software (Applied Biosystems, Foster City, California, USA). The results were obtained using the mathematical model Ratio = 2^-ΔΔct^ [43]. 72 h post transduction, puromycin was used for selecting positive colonies at final concentration 2 μg/ml. The sequences of the oligos used are found in S2 Table.

## Supporting information

S1 Fig Schematic of BifA’s conserved domains and its Fic domain sequence. BifA derived from SEZ ATCC35246 includes conserved RhuM and Fic domains. The amino acid sequence of BifA’s Fic domain is compared to that found in several pathogens. The position weight matrix was calculated by PWMEnrich package of R.
(TIF)

S2 Fig Growth of WT, ΔBif and CBif strains. The absorbance at OD_600_ of indicated strains cultured in THB media **(A)** or CFU derived from blood of i.v. infected mice **(B)** at the indicated times. All experiments were done in triplicate and means and SD are shown.
(TIF)

S3 Fig Appearance of intact brains and hematoxylin and eosin (H&E)-stained brain sections from mice inoculated with indicated SEZ strains. Mice were i.v. inoculated and sacrificed 2 days later. The arrows indicate blood vessels.
(TIF)

S4 Fig BifA concentration in bacterial culture supernatants. Immunoblots of supernatants from 2 independent overnight cultures of WT SEZ grown THB medium with anti-BifA antibody. Recombinant BifA protein was serially diluted to generate standard curves used to calculate BifA concentration in supernatant.
(TIF)

S5 Fig Addition of BifA to hBMEC monolayers decreases the amounts of ZO-1. The amount of ZO-1 detected by immunoblot with anti-ZO-1 antibody following addition of BifA (10 μg/ml) to hBMEC monolayers. The results shown are calculated from ZO-1 band intensities normalized to GAPDH band intensities shown in Fig 3D (measured with ImageJ software).
(TIF)

S6 Fig Co-purification of moesin with BifA from cell lysates. **hBMEC** cells were treated with either His-tagged BifA or BifA H247A for 2 hour. Then, the tagged BifA variants were purified on Ni+ columns. Moesin was detected by immunoblot in eluted samples from lysates of BifA but not BifA H247A treated cells.
(TIF)

S7 Fig Biacore analysis of BifA moesin interaction. **(A)** The Y-axis shows response units (RU), where 1 RU is equivalent to a change in surface protein concentration of 1 pg/mm. **(B)** Kinetic analysis of BifA and variants binding to phosphorylated or non-phosphorylated mutant moesin. Interaction kinetics are analyzed by monitoring the interaction as a function of time over a range of analyte concentrations (listed in the tables below sensorgrams). *K*_*D*_ values were calculated using the Biacore software.
(TIF)

S8 Fig Phos-tag detection of moesin phosphorylation in hBMEC. In all cases 10 ug of BifA or its variants were added to hBMEC monolayers at time 0 and moesin phosphorylation was monitored during the following 2 hr. The graphs shown on the right are gray scale intensity analyses (measured with ImageJ software).
(TIF)

S9 Fig BifA treatment leads to formation of GTP-Rac1. Western blots were performed on lysates of cells treated with BifA. Total Rac1 and rhotekin protein precipitated GTP-bound form Rac1 was detected using anti-Rac1 antibody. GAPDH was the loading control; the graph shown on the right are normalized gray scale intensity analyses (measured with ImageJ software).
(TIF)

S10 Fig Moesin depletion in hBMEC cells treated with lentivirus delivered siRNA targeting moesin. Moesin transcript (qPCR) **(A)** and protein (immunoblot) levels **(B)** in hBMEC cells after infection with lentivirus encoded interfering RNA targeting moesin; **(C)** Immunofluorescence of moesin in WT and moesin knock-down hBMECs.
(TIF)

S11 Fig Schematic model of BifA disruption of endothelial cells in the BBB. SEZ (blue chains) releases BifA (blue rectangle with yellow strip representing the Fic domain), which enters into brain endothelial cells. BifA binds to the moesin ERMAD domain and promotes its PKC-dependent phosphorylation, which may lead to a change in moesin conformation [16]. Activation of moesin leads to formation of RhoA-GTP, which promotes loosening of tight junctions. These changes disrupt the integrity of the endothelial cell barrier facilitating SEZ entry into the brain.
(TIF)

S1 Table SILAC identified HEK293T proteins which had potential function of interaction with BifA. (XLSX)

S2 Table Strains, plasmids, and primers used in this study. (XLSX)

S1 Movie Appearance of BifA treated hBMEC monolayers. The red number in the left corner indicates time (minute). Arrows indicate gaps formation positions.
(AVI)

S2 Movie Appearance of BifA treated hBMEC monolayers. The red number in the left corner indicates time (minute). Arrows indicate gaps formation positions.
(AVI)

S3 Movie Appearance of BifA H247A mutant treated hBMEC monolayers. The red number in the left corner indicates time (minute). Arrows indicate gaps formation positions.
(AVI)

S4 Movie Appearance of BifA H247A mutant treated hBMEC monolayers. The red number in the left corner indicates time (minute). Arrows indicate gaps formation positions.
(AVI)

S5 Movie Appearance of DMEM (mock) treated hBMEC monolayers. The red number in the left corner indicates time (minute). Arrows indicate gaps formation positions.
(AVI)

S6 Movie Appearance of DMEM (mock) treated hBMEC monolayers. The red number in the left corner indicates time (minute). Arrows indicate gaps formation positions.
(AVI)
