# Supplementary figures and images for: A streptococcal Fic domain-containing protein disrupts blood-brain barrier integrity by activating moesin in endothelial cells
Source: PLoS Pathog. 2019 May 9;15(5):e1007737. doi: 10.1371/journal.ppat.1007737 (PMC6529018; doi:10.1371/journal.ppat.1007737)

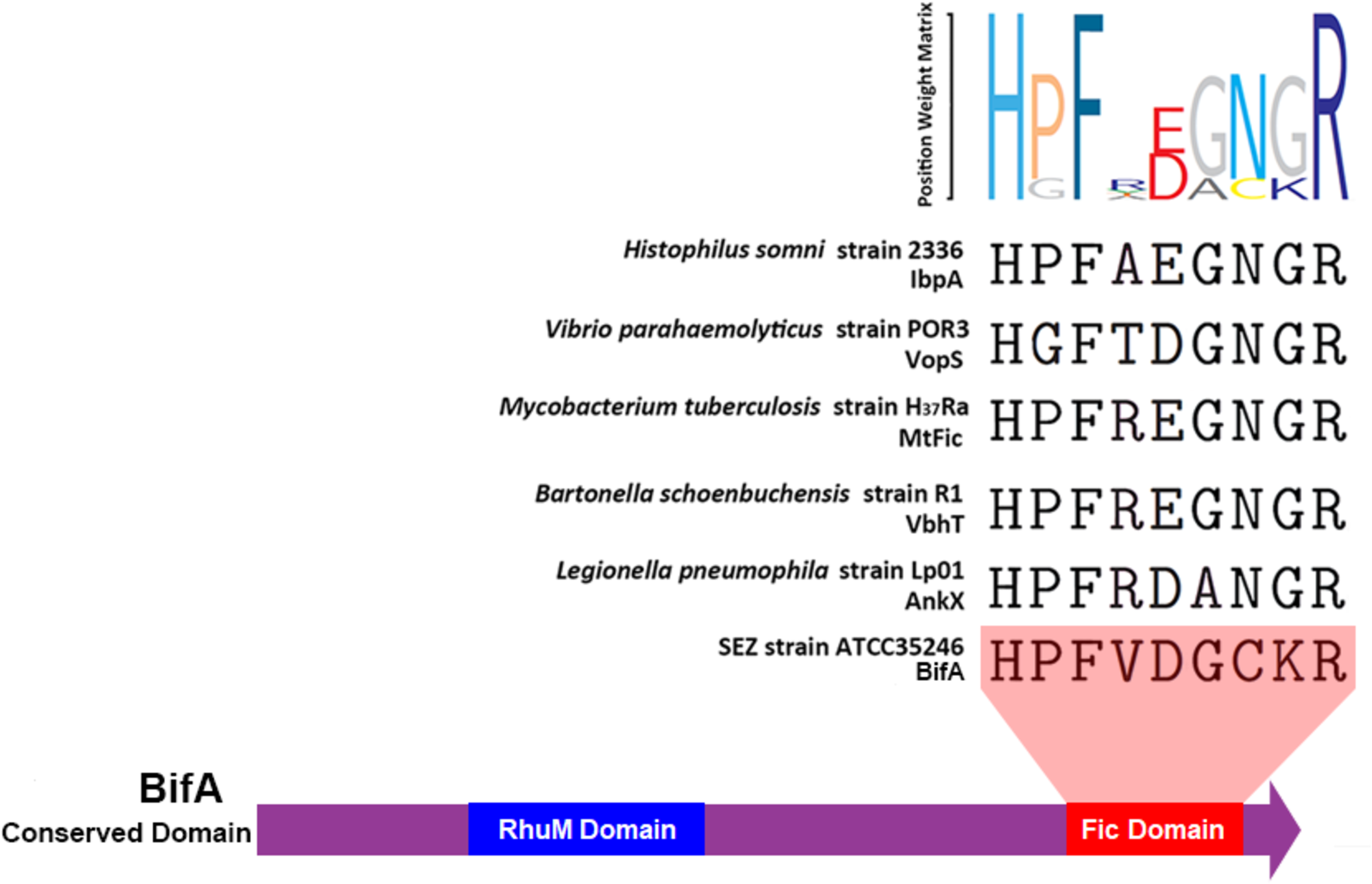

Supplement: S1 Fig — BifA derived from SEZ ATCC35246 includes conserved RhuM and Fic domains. The amino acid sequence of BifA’s Fic domain is compared to that found in several pathogens. The position weight matrix was calculated by PWMEnrich package of R. (TIF) [file ppat.1007737.s001.tif]

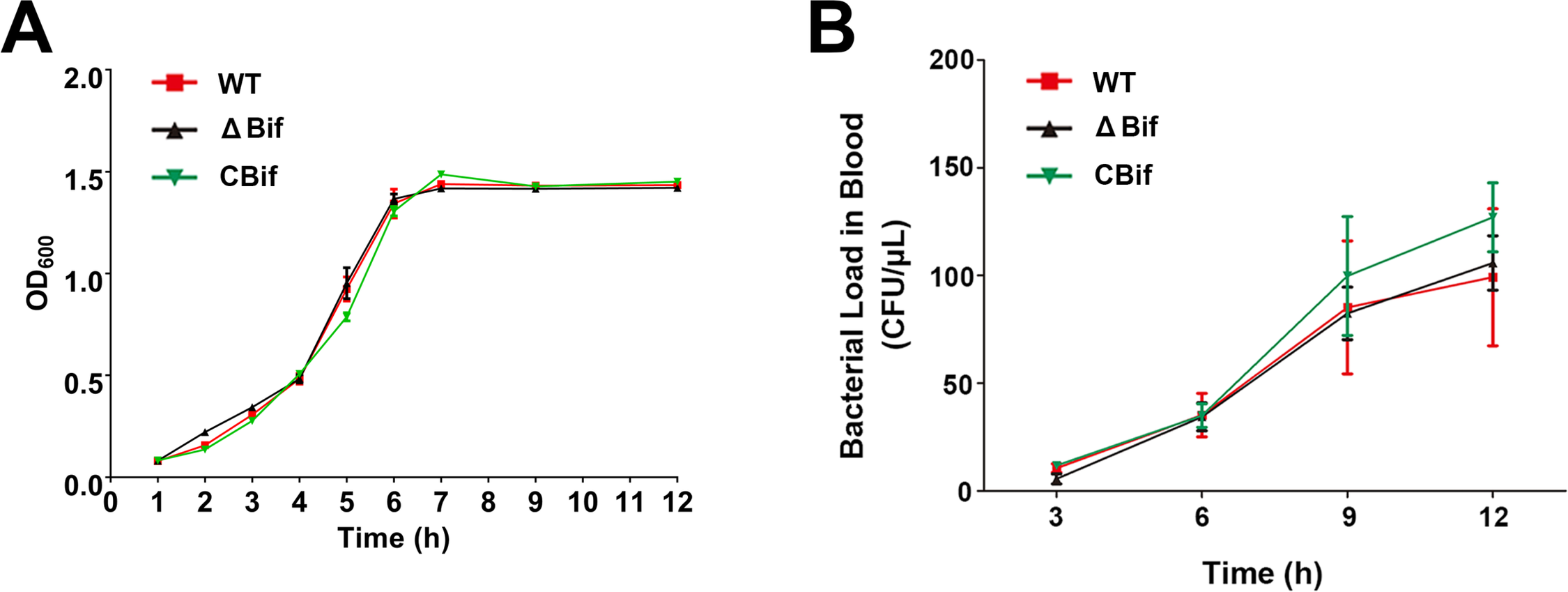

Supplement: S2 Fig — The absorbance at OD600 of indicated strains cultured in THB media (A) or CFU derived from blood of i.v. infected mice (B) at the indicated times. All experiments were done in triplicate and means and SD are shown. (TIF) [file ppat.1007737.s002.tif]

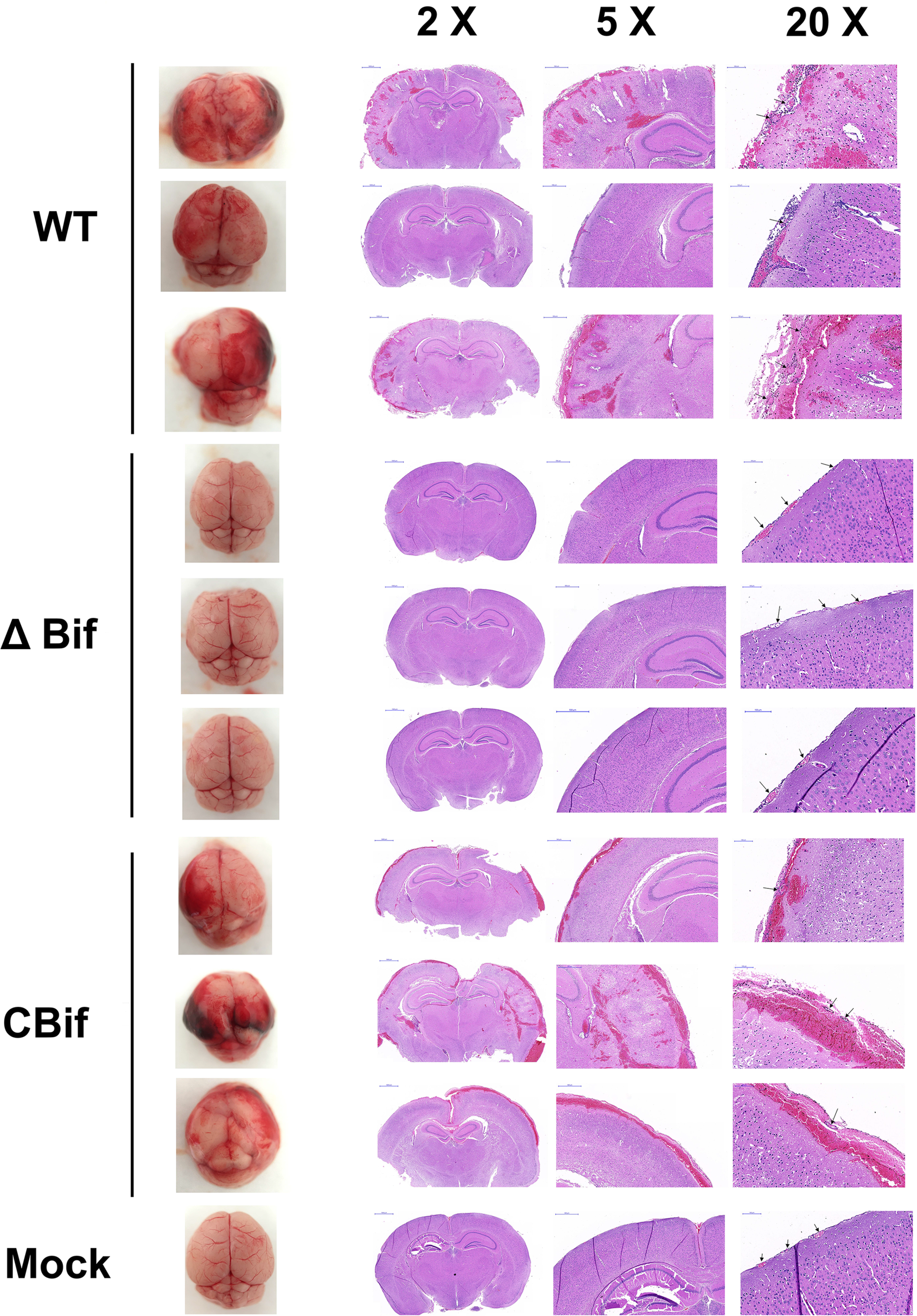

Supplement: S3 Fig — Mice were i.v. inoculated and sacrificed 2 days later. The arrows indicate blood vessels. (TIF) [file ppat.1007737.s003.tif]

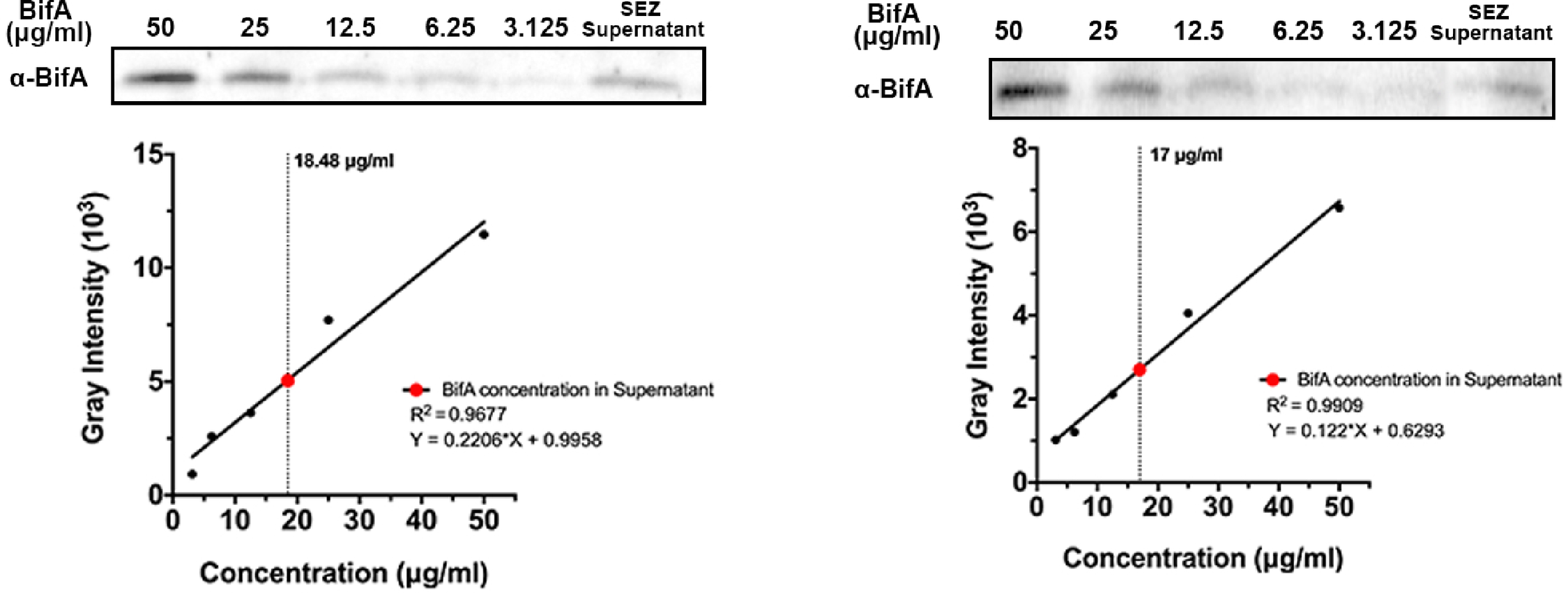

Supplement: S4 Fig — Immunoblots of supernatants from 2 independent overnight cultures of WT SEZ grown THB medium with anti-BifA antibody. Recombinant BifA protein was serially diluted to generate standard curves used to calculate BifA concentration in supernatant. (TIF) [file ppat.1007737.s004.tif]

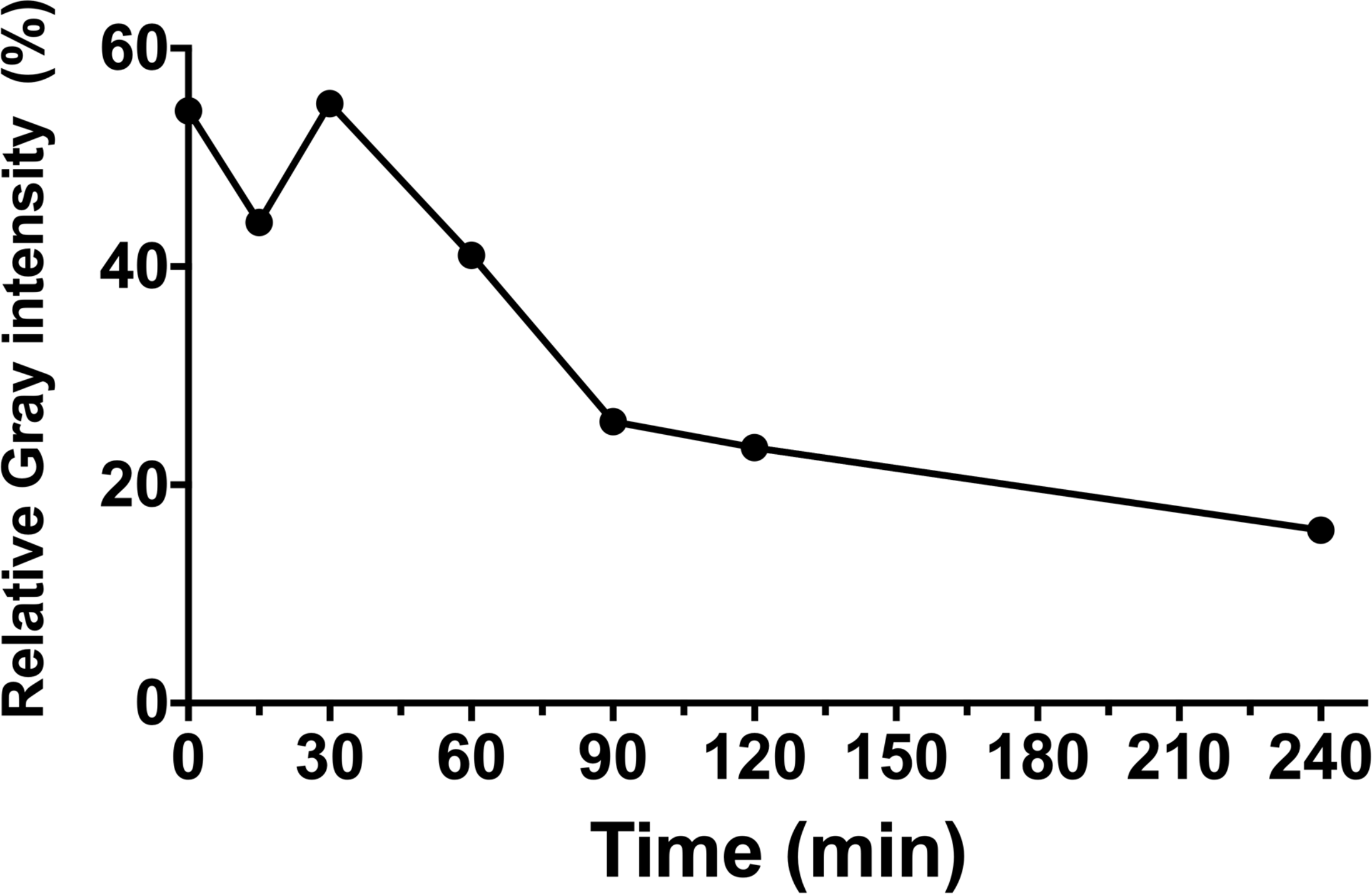

Supplement: S5 Fig — The amount of ZO-1 detected by immunoblot with anti-ZO-1 antibody following addition of BifA (10 μg/ml) to hBMEC monolayers. The results shown are calculated from ZO-1 band intensities normalized to GAPDH band intensities shown in Fig 3D (measured with ImageJ software). (TIF) [file ppat.1007737.s005.tif]

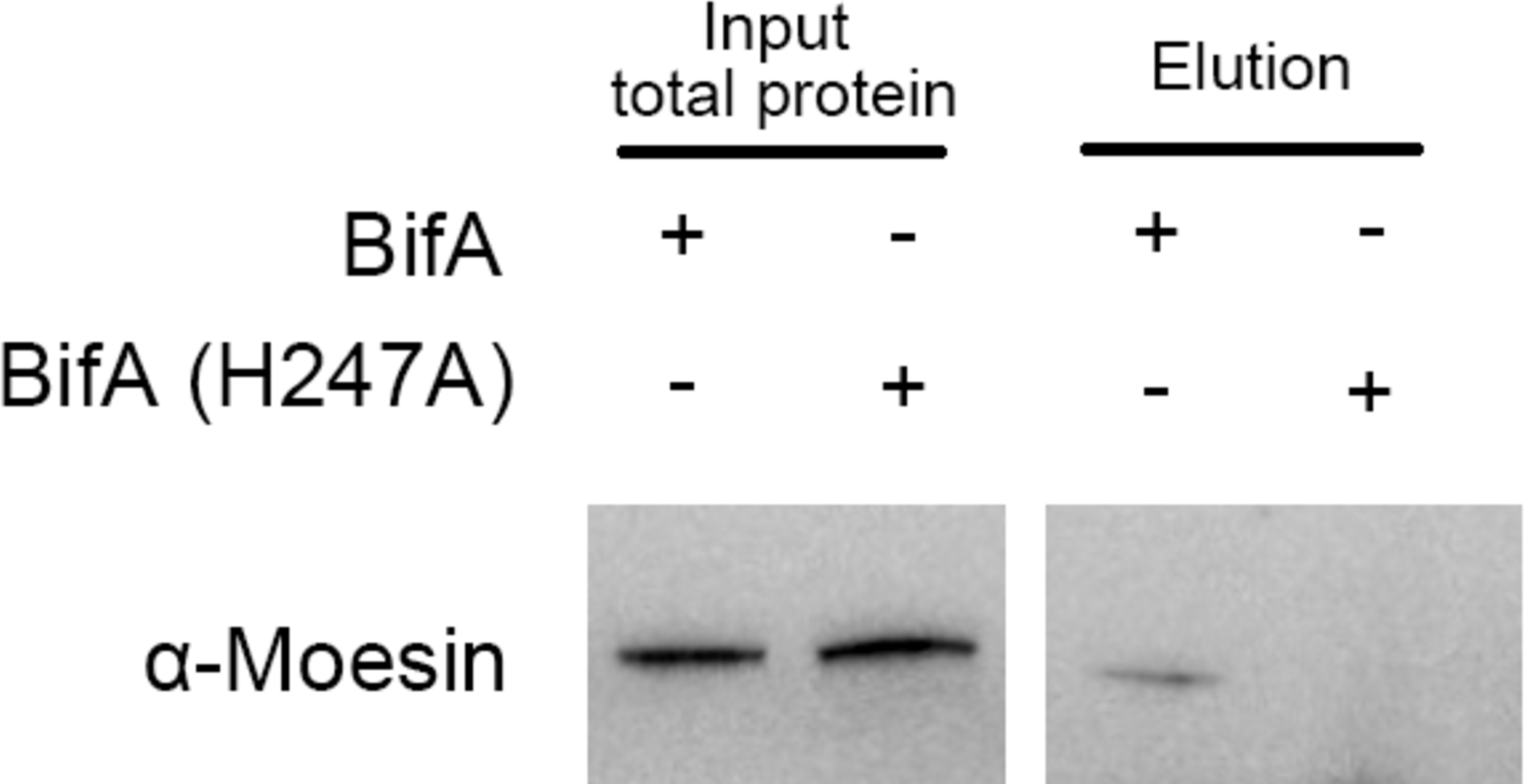

Supplement: S6 Fig — hBMEC cells were treated with either His-tagged BifA or BifA H247A for 2 hour. Then, the tagged BifA variants were purified on Ni+ columns. Moesin was detected by immunoblot in eluted samples from lysates of BifA but not BifA H247A treated cells. (TIF) [file ppat.1007737.s006.tif]

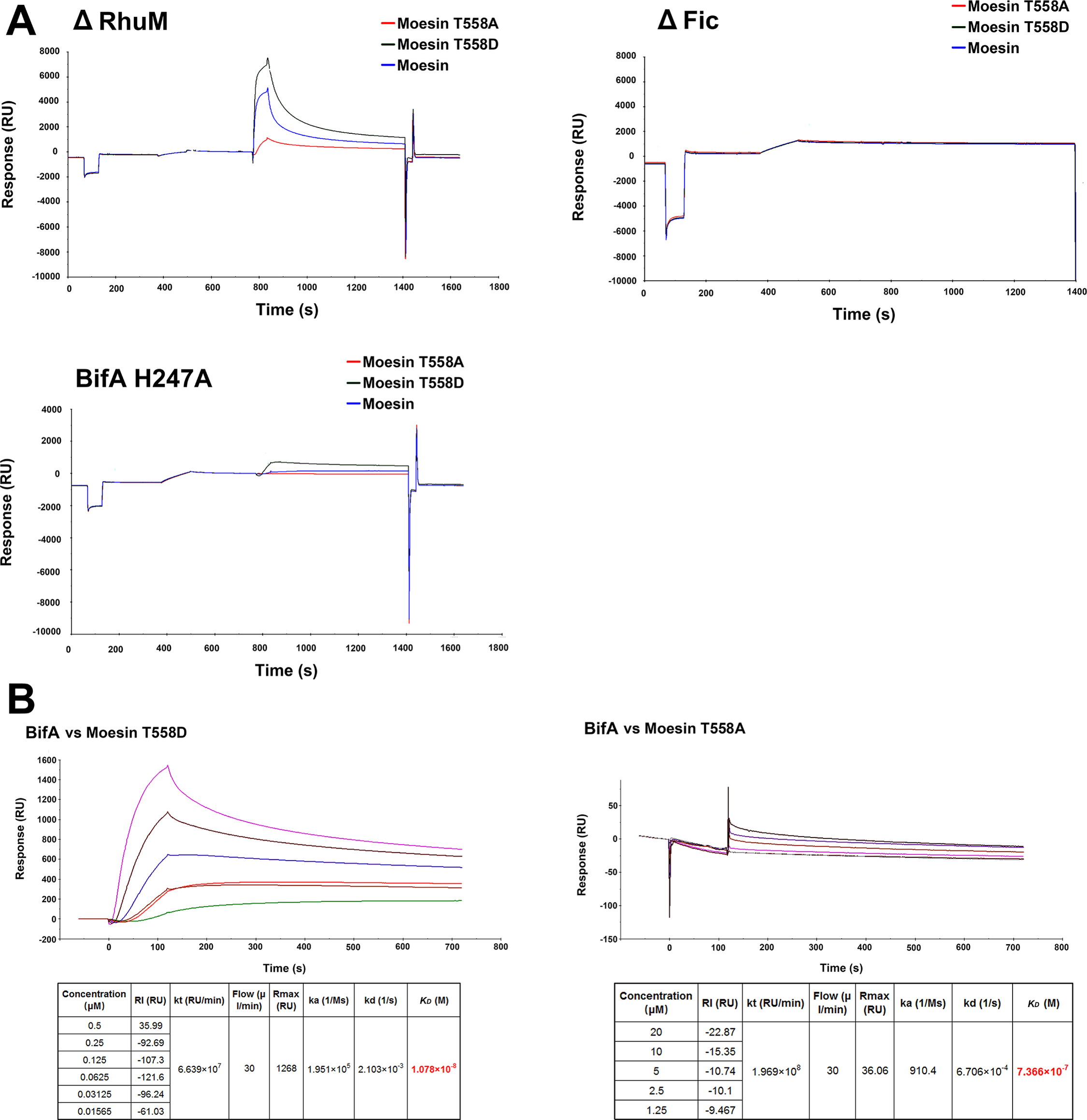

Supplement: S7 Fig — (A) The Y-axis shows response units (RU), where 1 RU is equivalent to a change in surface protein concentration of 1 pg/mm. (B) Kinetic analysis of BifA and variants binding to phosphorylated or non-phosphorylated mutant moesin. Interaction kinetics are analyzed by monitoring the interaction as a function of time over a range of analyte concentrations (listed in the tables below sensorgrams). KD values were calculated using the Biacore software. (TIF) [file ppat.1007737.s007.tif]

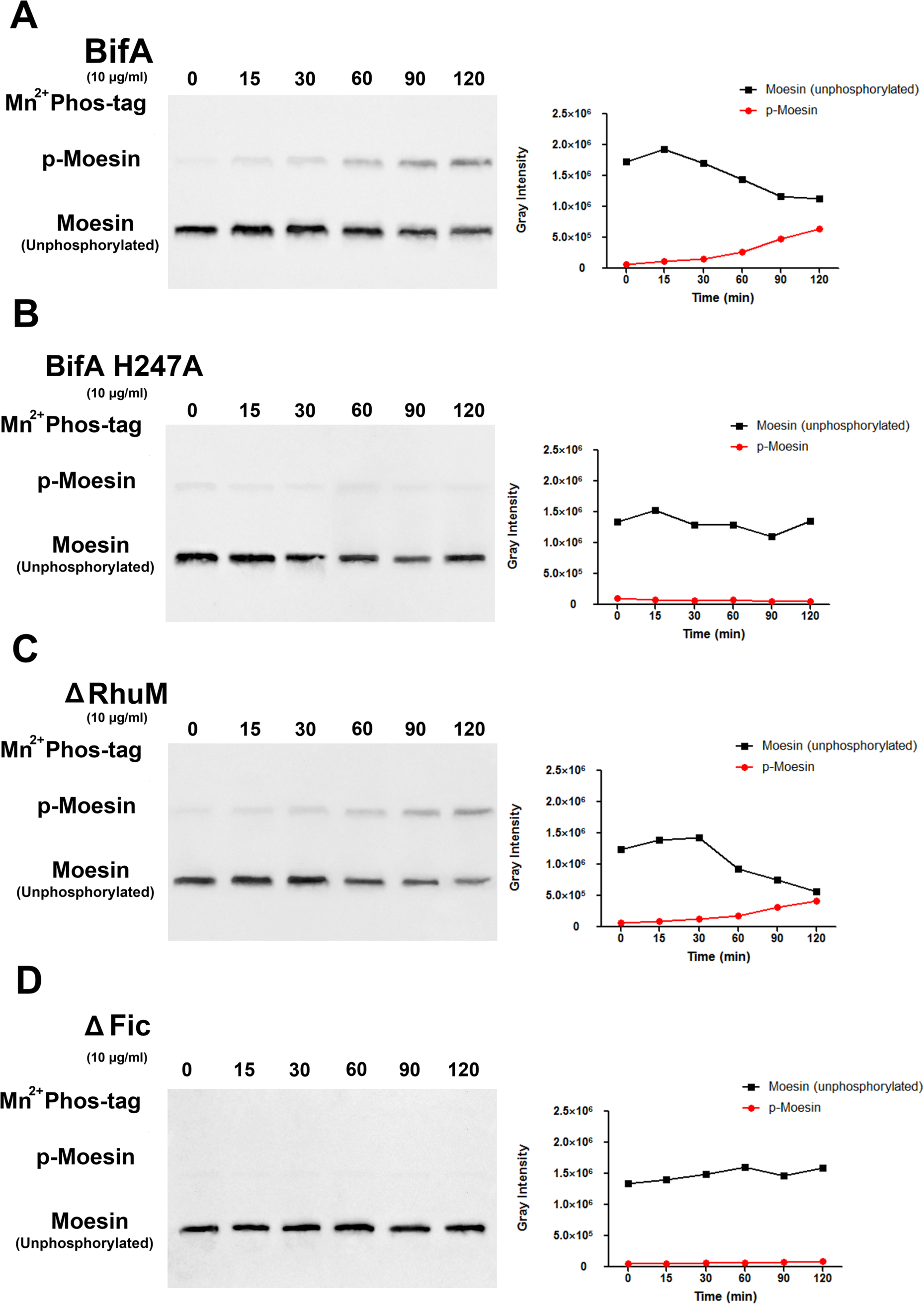

Supplement: S8 Fig — In all cases 10 ug of BifA or its variants were added to hBMEC monolayers at time 0 and moesin phosphorylation was monitored during the following 2 hr. The graphs shown on the right are gray scale intensity analyses (measured with ImageJ software). (TIF) [file ppat.1007737.s008.tif]

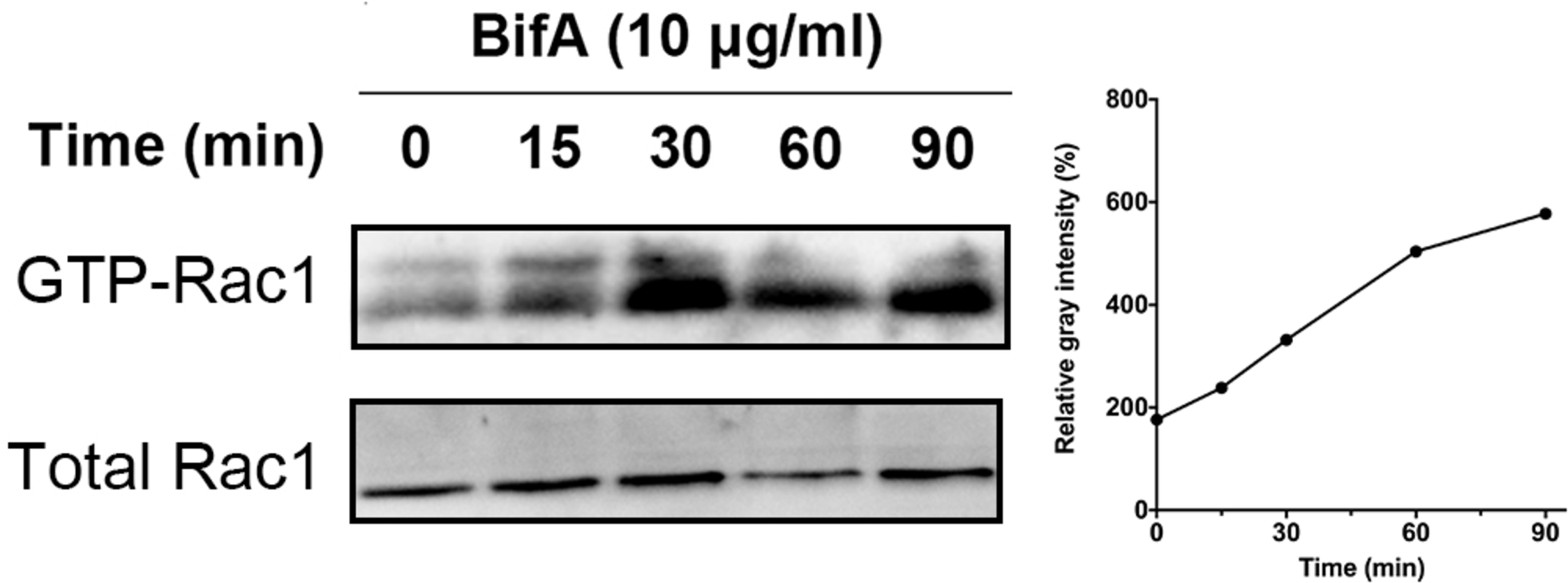

Supplement: S9 Fig — Western blots were performed on lysates of cells treated with BifA. Total Rac1 and rhotekin protein precipitated GTP-bound form Rac1 was detected using anti-Rac1 antibody. GAPDH was the loading control; the graph shown on the right are normalized gray scale intensity analyses (measured with ImageJ software). (TIF) [file ppat.1007737.s009.tif]

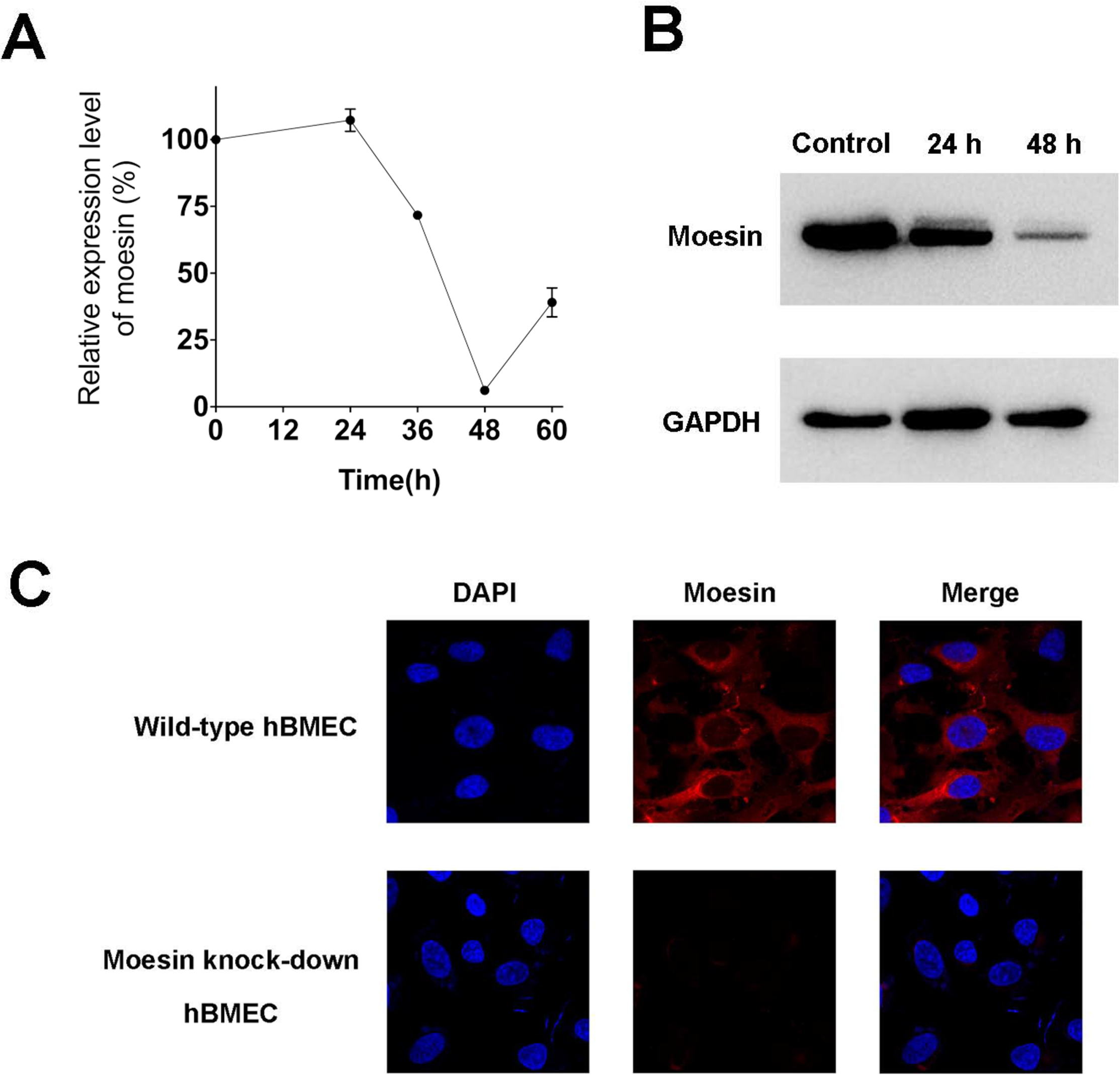

Supplement: S10 Fig — Moesin transcript (qPCR) (A) and protein (immunoblot) levels (B) in hBMEC cells after infection with lentivirus encoded interfering RNA targeting moesin; (C) Immunofluorescence of moesin in WT and moesin knock-down hBMECs. (TIF) [file ppat.1007737.s010.tif]

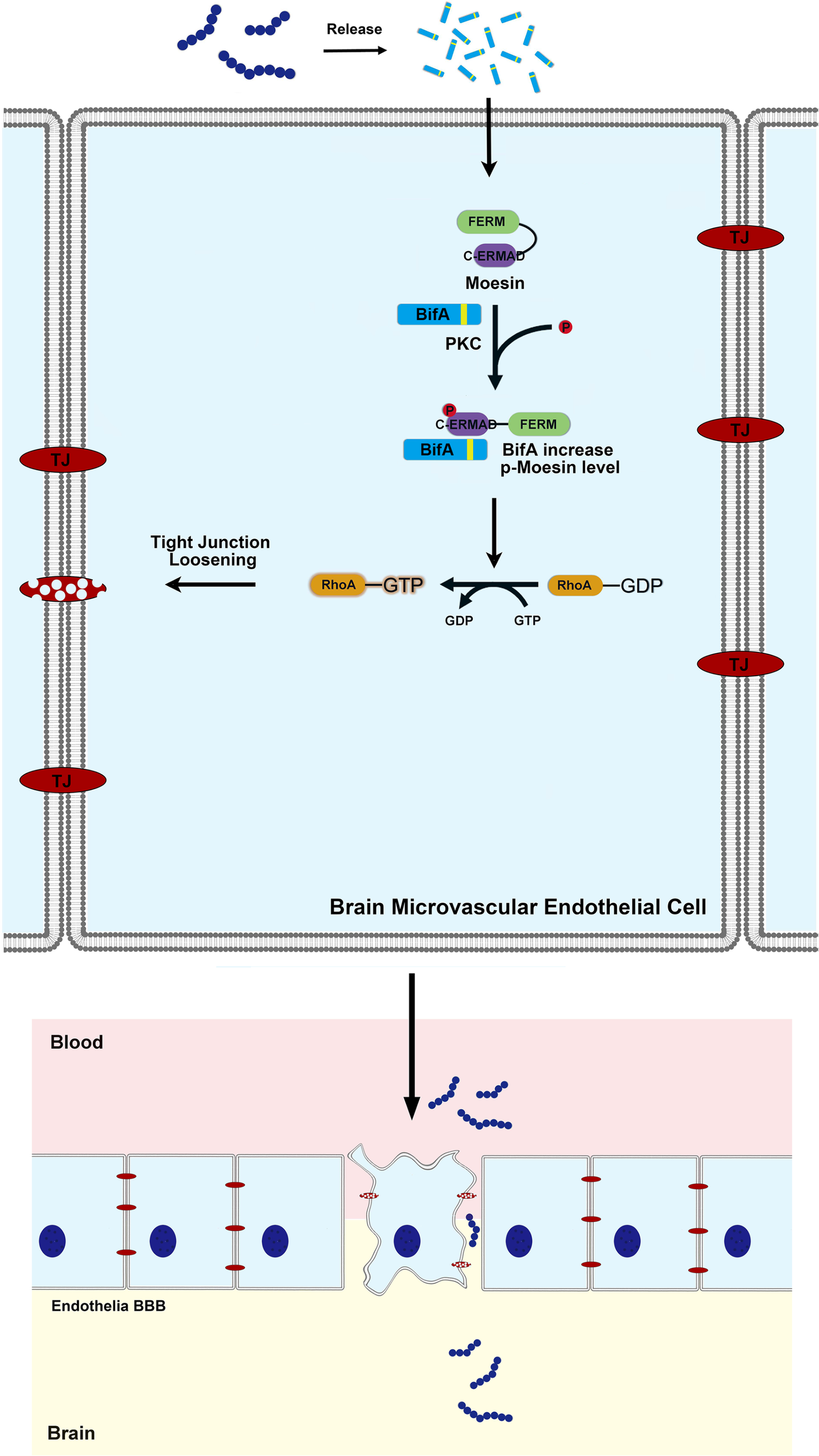

Supplement: S11 Fig — SEZ (blue chains) releases BifA (blue rectangle with yellow strip representing the Fic domain), which enters into brain endothelial cells. BifA binds to the moesin ERMAD domain and promotes its PKC-dependent phosphorylation, which may lead to a change in moesin conformation [16]. Activation of moesin leads to formation of RhoA-GTP, which promotes loosening of tight junctions. These changes disrupt the integrity of the endothelial cell barrier facilitating SEZ entry into the brain. (TIF) [file ppat.1007737.s011.tif]
